# Slow Water in Engineered Nanochannels Revealed by Color-Center-Enabled Sensing

**DOI:** 10.1021/acs.nanolett.5c01344

**Published:** 2025-06-12

**Authors:** Daniela Pagliero, Rohma Khan, Kapila Elkaduwe, Ankit Bhardwaj, Kang Xu, Abraham Wolcott, Gustavo E. López, Boya Radha, Nicolás Giovambattista, Carlos A. Meriles

**Affiliations:** † Department of Physics, 14770 CUNY—The City College of New York, New York, New York 10031, United States; ‡ CUNY-Graduate Center, New York, New York 10016, United States; § Department of Physics, CUNY—Brooklyn College of the City University of New York, Brooklyn, New York 11210, United States; ∥ Department of Physics and Astronomy, 5292 The University of Manchester, Manchester M13 9PL, U.K.; ⊥ Photon Science Institute and National Graphene Institute, 5292 University of Manchester, Manchester M13 9PL, U.K.; # Department of Chemistry, San José State University, San José, California 95192, United States; ∇ Department of Chemistry, CUNY—Lehman College, Bronx, New York 10468, United States

**Keywords:** confined water, quantum sensing, NV centers, nanochannels, H_2_O self-diffusion

## Abstract

Nanoscale confinement of liquids can result in enhanced viscosity, local fluidic order, or collective motion. Studying these effects, however, is notoriously difficult, mainly due to the lack of experimental methods with the required sensitivity and spatial or time resolution. Here we leverage shallow nitrogen-vacancy (NV) centers in diamond to probe the dynamics of room-temperature water molecules entrapped within ∼5 nm-tall channels formed between the diamond crystal and a suspended hexagonal boron nitride (hBN) flake. NV-enabled nuclear magnetic resonance measurements of confined water protons reveal a much reduced H_2_O self-diffusivity, orders of magnitude lower than that in bulk water. We posit the slow dynamics stem from the accumulation of photogenerated carriers at the interface and trapped fluid, a notion we support with the help of molecular dynamics modeling. Our results expose the importance of space charge fields in theories describing interfacial water and lay out a route for investigating other fluids under confinement.

## Graphical abstract

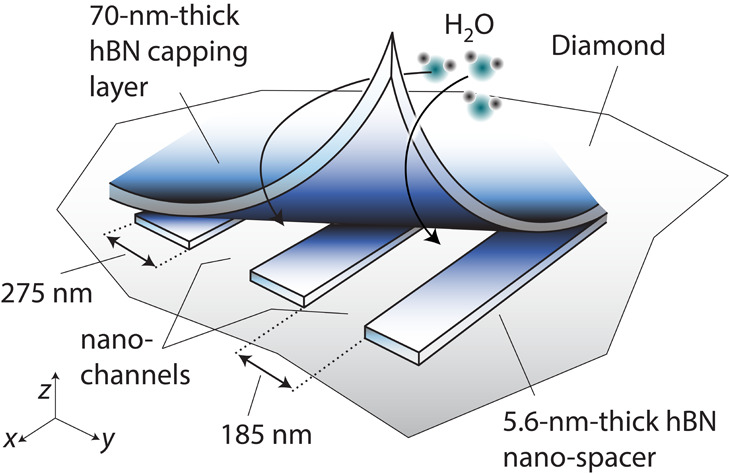

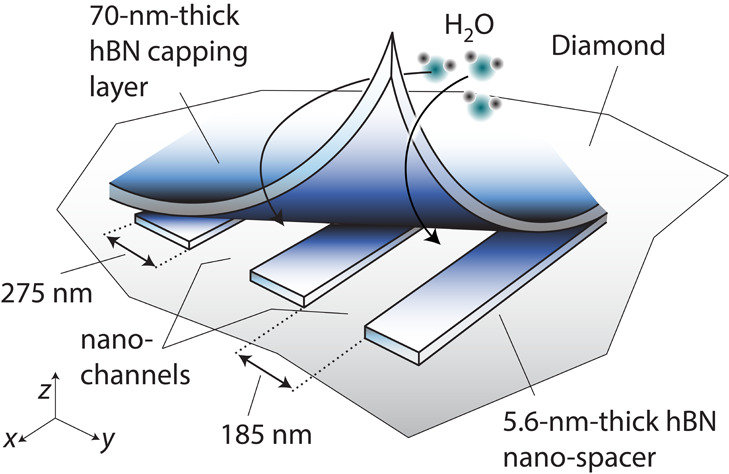

Liquids confined to small volumes are presently the subject of much interest as the complex dynamics they display have broad implications in areas spanning geophysics, tribology, catalysis, polymer science, and biology.^1–3^ To probe the molecular dynamics in these systems, experimenters have resorted to various techniques including electrochemical and optical methods, photoelectron and electron spectroscopies, ion and neutron scattering, field emission microscopy, and X-ray or electron diffraction, to mention just some.^4^ Out of these, however, only a few have the combined spatial and temporal sensitivity to probe molecular ensembles locally. For example, recent work has demonstrated the use of atomic force microscopy (AFM) to examine the rheology of liquids confined between the apex of the AFM tip and a solid substrate.^5–8^ Unfortunately, AFM requires direct access to the sample and ranks as an invasive technique with poor time resolution, ill-adapted for sealed fluidic systems or for probing dynamical processes.

Here, we demonstrate the combined use of quantum sensing and 2D materials engineering to physically confine and probe the molecular dynamics of water, a notion proposed theoretically^9^ but not yet realized. To circumvent the sensitivity limitations inherent to inductively detected nuclear magnetic resonance (NMR), we make use of a small ensemble of near-surface nitrogen-vacancy (NV) centers as optically detected magnetic probes. Sensitive to water mobility, our ^1^H NMR experiments reveal H_2_O self-diffusivity orders of magnitude below that found in bulk water. Molecular dynamics (MD) simulations indicate confinement alone is insufficient to account for the observed phenomenology and support instead the notion of an electric-field-induced process resulting from the interfacial accumulation of photogenerated carriers. These findings relate to work by other groups reported during the writing of this article.^10,11^

## ^1^H NMR of Confined Water

Figure 1a shows a schematic of the nanochannel structure.^12–14^ We use electron beam lithography to pattern a set of 5.6 nm-tall strips of hBN, which we then cap with a hBN flake sufficiently thick (70 nm) to avoid sagging;^15^ windows on either end of the channels serve as inlet/exit ports (Figures 1b and 1c). To gain control over the environment, we enclose the structure in a microfluidic chip and fill in the nanochannels by exposure to saturated water vapor (Supporting Information (SI), Section I).

**Figure 1:**
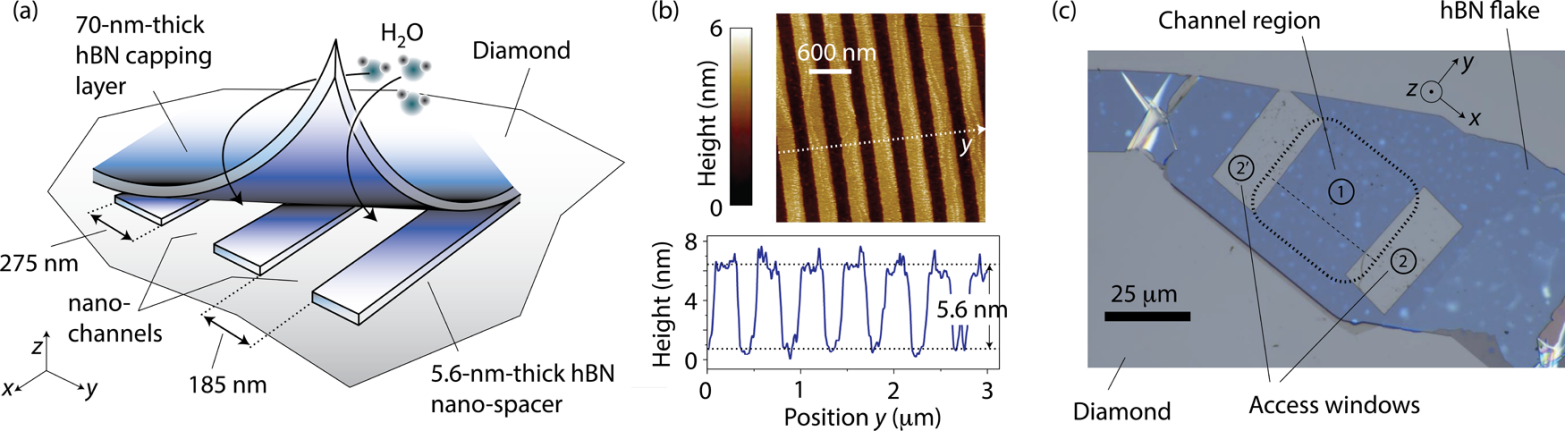
**Nanoscale confinement of water molecules**. (a) Schematic of the nanochannel structure. We overlay an ∼70 nm-thick hBN flake on a set of 5.6 nm-tall hBN spacers previously engineered on the surface of a [100] diamond via electron beam lithography. The crystal sits within a sealed chamber in a microfluidic device (not shown), hence allowing us to control the sample environment. (b) Atomic force microscopy image of a channel section prior to adding the capping hBN layer; the bottom inset is a cross-sectional plot across the dashed line. (c) Scanning electron microscopy image of the complete structure. The dashed square (here denoted as area ①) indicates the nanochannel section; the dashed line runs parallel to the channels, and water intake takes place through two rectangular side openings (areas ②).

We combine chemical vapor deposition overgrowth, ^15^N implantation, and thermal annealing to cap a starting seed diamond with a ^13^C-depleted layer hosting ∼8 nm-deep NV centers, here acting as nanoscale spin sensors^16^ (Figure 2a). For the present experiments, we apply a magnetic field in the 38–45 mT range along one of the four possible crystalline NV axes and tune the microwave (MW) from a nearby antenna to the |*m*_NV_ = 0⟩ ↔ |*m*_NV_ = −1⟩ NV spin transition.^17^ For illustration, the plot in Figure 2b shows an optically detected magnetic resonance (ODMR) NV spectrum, manifesting as an asymmetric doublet due to nuclear spin pumping of the ^15^N host (SI, Section I).

**Figure 2:**
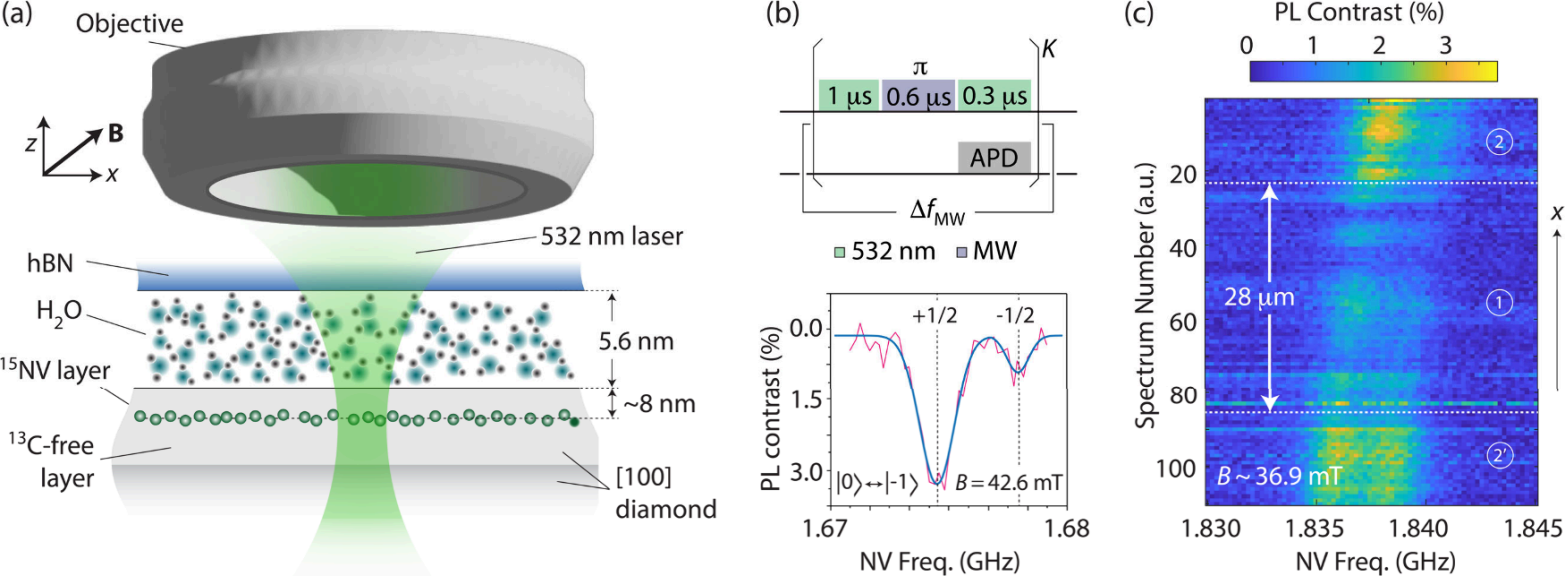
**Nanoscale sensing via shallow NV centers**. (a) We implement nanoscale ^1^H NMR with the help of an 8 nm-deep layer of engineered ^15^NV centers serving as magnetic sensors. (b) (Top) Pulsed ODMR protocol; we average the NV PL after *K* repeats of a MW π-pulse, whose frequency we vary in steps Δ*f*_MW_ to reconstruct the NV spin resonance spectrum. (Bottom) ODMR of the |*m*_NV_ = 0⟩ ↔ |*m*_NV_ = −1⟩ ^15^NV transition. The vertical dashed lines mark the frequencies expected for the PL dips corresponding to the *m*_N_ = ±1/2 projections of the hyperfine coupled ^15^N host, and the solid trace is a Gaussian fit. Careful alignment of the externally applied magnetic field **B** along one of the crystalline NV axes—at 54.7° relative to the crystal normal *z*—leads to near-full ^15^N spin polarization. (c) NV ODMR along a line parallel to the channels (area ①) and connecting the two access windows (areas ②, see dashed line in Figure 1c). Notably, hBN partially quenches the NV spin contrast. APD: Avalanche photo detector. MW: Microwave. Freq: Frequency.

NV sensing in the nanochannel area is challenging, because the hBN structure has a detrimental impact on the NV spin and optical properties. Figure 2c shows the NV ODMR spectrum at different positions along a line connecting the access windows (dashed line in Figure 1c): Besides the slight frequency shift and changing amplitude ratio in the resonance doublet (a consequence of magnetic field heterogeneity), we observe a reduction in the photoluminescence (PL) contrast. This behavior is a likely consequence of surface-induced NV charge dynamics, a subject we return to later (see also SI, Sections I and II).

To measure the ^1^H NMR spectrum from water, we implement a dynamical decoupling sensing protocol comprising a train of MW π-pulses separated by a time interval τ (Figure 3a). For long pulse trains, NVs become selectively sensitive to magnetic fields oscillating within a narrow frequency window centered at 1/2τ.^18^ Correspondingly, a signature NMR signal develops as one varies the interpulse spacing to match half the ^1^H spin precession period at the externally applied magnetic field.^19,20^ Figure 3a shows representative examples before and after the channels are exposed to saturated water vapor: We observe a clear PL dip at the ^1^H Larmor frequency, hence revealing the presence of entrapped water. Slow evaporation gradually empties the channels over a time span of 2 to 3 weeks (left panel in Figure 3a and SI, Section I), which allows us to recycle the confining structure for repeated use. This trait is advantageous here as it lifts ambiguities on the nature of the sensed molecules, potentially a complication in fully encapsulated geometries.

**Figure 3:**
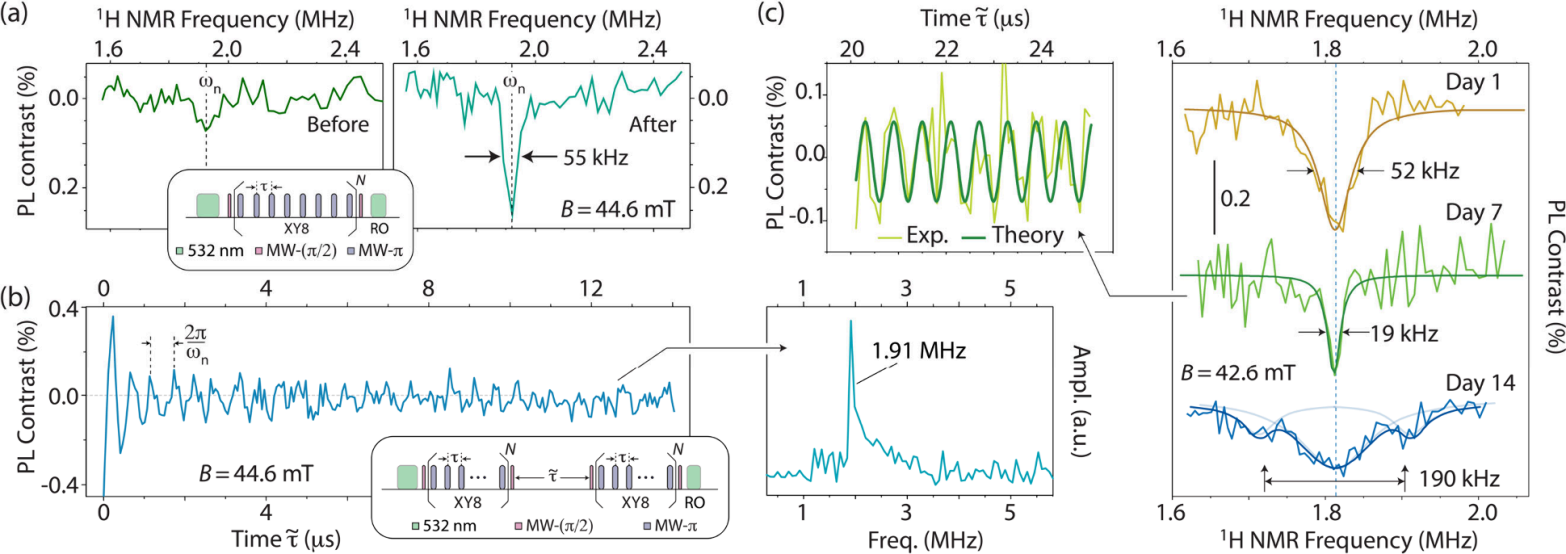
^**1**^ **H NMR spectrum of confined water**. (a) ^1^H NMR spectra before and after filling the channels with water (left- and right-hand side plots, respectively). The vertical dashed line indicates the expected ^1^H Larmor frequency ω_*n*_. The small dip prior to water exposure is likely a leftover from prior fillings. We use *N* = 10 XY8 decoupling units and apply 4 × 10^6^ repeats for each interpulse interval τ. (b) ^1^H spin correlation signal after channel filling and corresponding Fourier transform (right-hand side insert). The conditions are those of (a), except that *N* = 5. (c) (Right panel) NV-detected ^1^H NMR spectra of confined water at different times (1, 7, and 14 days) after filling the channels. Smooth solid traces are Lorentzian fits, and the vertical dashed line indicates the expected ^1^H Larmor frequency ω_*n*_. (Left panel) ^1^H correlation signal 1 week after channel filling (green trace in the main panel); the solid trace is sinusoidal at the ^1^H Larmor frequency. We observe a nuclear spin coherence exceeding 25 μs of free evolution with no apparent decay. RO: Readout. Ampl.: Signal amplitude.

## Water Diffusion under Nanochannel Confinement

The ability to probe unpolarized water protons via NV centers—to our knowledge, shown here for the first time—is intriguing because H_2_O molecules leave the NV sensing range *d* ∼ 8 nm within a time *T*_D_^(b)^ ∼ *d* ^2^/6*D* ^(b)^; using the self-diffusion coefficient of bulk water *D* ^(b)^ = 2.3 × 10^–9^ m^2^ s^–1^ as a reference,^21^ we find *T*_D_^(b)^ ∼ 5 ns, orders of magnitude shorter than the μs-duration of the MW pulse trains required for nuclear spin sensing.

To gauge the diffusion dynamics of water more accurately, we implement a spin correlation measurement whose output reflects on the ^1^H spin coherence after a variable evolution time τ̃.^22^ Molecular diffusion during this interval amounts to a phase disruption in the nuclear spin set being sensed and thus leads to a reduction in the NV signal amplitude. Because decoherence from internuclear dipolar couplings is weak for a fluid, molecular self-diffusion defines the characteristic signal decay time.^23,24^

Figure 3b shows the results: We observe a distinctive oscillatory response centered at the ^1^H Larmor frequency whose envelope features a rapid initial decay followed by a long-lived tail, a consequence of the single-sided geometry of NV center sensing.^24^ A detailed analysis reveals a bimodal envelope with fast and slow time constants *T*_D_^(f)^ = 0.5 μs and *T*_D_^(s)^ = 30 μs (SI, Section II). In either case, we derive diffusion constants *D* ^(f)^ ≈ *d* ^2^/6*T*_D_^(f)^ ∼ 2.1 × 10^–11^ m^2^ s^–1^ and *D* ^(s)^ ∼ 0.4 × 10^–12^ m^2^ s^–1^, orders of magnitude lower than in bulk water.

We caution that there is substantial variability in the evolution of the spectra after channel filling, which suggests complex dynamics, possibly impacted by uneven evaporation over time. For example, the right panel in Figure 3c showcases an instance where the NMR line width (52 kHz at the time of the channel refill, yellow trace) shrinks to less than half the original value after a week-long interval (green trace). Correlation measurements at that time revealed ^1^H coherences extending over tens of microseconds with minimal decay; from the trace in the left panel of Figure 3c, we derive *T*_D_ ≥ 100 μs, and correspondingly *D* ≤ 0.1 × 10^–12^ m^2^ s^–1^.

Subsequent observations after a two week span showed a much broader NV-NMR spectrum whose reduced amplitude prevented correlation measurements (blue trace in Figure 3c). Interestingly, the weak satellites flanking the main dip suggest this broadening may not necessarily stem from faster molecular diffusion. In particular, the magnitude of this splitting (largely exceeding that reported for water protons on silica^25^) hints at couplings with electronic spins.^26^ Overall, this time-dependent behavior is reminiscent of driven, dissipative systems that are able to reach a statistically stationary distribution while lacking a well-defined instantaneous configuration (see below).

To rationalize these observations, we posit that the suppressed water diffusivity arises from confinement-assisted accumulation of interfacial charge, driven by photoinduced carrier injection from the surrounding walls. In diamond, such injection can occur under green (532 nm) excitation via photoionization of near-surface defect states (e.g., NV centers or other deep levels), even though the excitation is sub-bandgap. This process is particularly favorable in hydrogen-terminated diamond^27,28^ due to its large negative surface electron affinity, although it persists in acid-cleaned surfaces (featuring remnant hydroxyl and CH groups^29^), especially when in contact with water.^30^ Specifically, prior work^31^ in similar NV-doped diamond surfaces shows that shallow defects (including NVs) release carriers into adjacent aqueous environments as they charge cycle under green illumination;^32^ moreover, we would expect similar processes to occur at the hBN/liquid interface. We hypothesize that this carrier injection leads to electric double layer formation, enhancing molecular self-organization and slowing H_2_O dynamics near the confining surfaces. Note that, in the presence of gradual evaporation, one would anticipate a dynamic charge buildup process and hence fluctuations of space charge fields over time, consistent with our observations.

## Molecular Dynamics Simulation of Confined Water

As a starting reference, we first study the dynamics of pure water confined to a geometry emulating our experimental conditions (Figure 4a). Given the compositional heterogeneity of chemically oxidized diamond surfaces,^33^ we consider four different terminations^34^ (H, OH, O, and H–OH, see SI, Section III). The top panel in Figure 4b displays the calculated water density profile between the confining walls of the hydroxylated diamond surface. Similar to prior experiments^35–37^ and MD simulations,^38^ proximity to the walls leads to the formation of discrete molecular layers—L1 through L3—but the order extends only over distances of ∼1 nm. Further, from the bottom plot in Figure 4b we derive mean-square displacements (MSDs) exceeding 60 nm^2^ within 6 ns, thus indicating that water molecules leave the sensing volume with rates characterized by bulk-like diffusion coefficients. It follows that MD simulations of nanoconfined (pure) water are *incompatible* with the observed NV-NMR signal.

**Figure 4:**
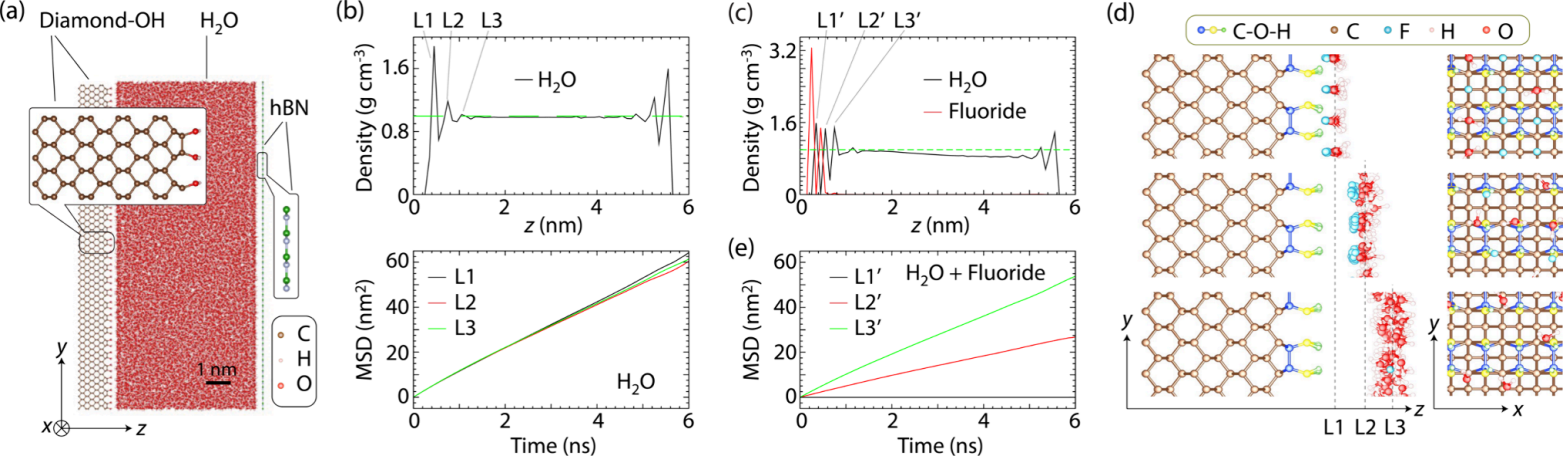
**Molecular dynamics simulations of confined water with and without added charges**. (a) Snapshot from MD simulations of a system comprising *N*_w_ = 30442 water molecules confined by an hBN monolayer and a hydroxylated diamond at a temperature *T* = 300 K. (b) (Top) Water density profile as a function of the distance *z* from the diamond surface for the system shown in (a). (Bottom) Time dependence of the mean-square displacement (MSD) for water molecules initially within layers L1 (*z* < 0.55 nm), L2 (0.55 nm < *z* < 0.95 nm), and L3 (0.95 nm < *z* < 1.25 nm) next to the diamond crystal. (c) Calculated water and fluoride density profiles as a function of the distance *z* from the diamond surface after replacing *N*_q_ = 2704 water molecules in (a) by fluoride anions; we attain charge neutrality by distributing positive charges in the diamond (SI, Section III). The green dashed line indicates the bulk density of water under ambient conditions. (d) Side and top views of layers L1′–L3′ defined in (c). (e) MSD as a function of time for water molecules initially located within layers L1′ (*z* < 0.45 nm), L2′ (0.45 nm < *z* < 0.65 nm), and L3′ (0.65 nm < *z* < 0.85 nm). Molecules within L2′ and L3′ (respectively, red and green traces) diffuse away, while molecules in L1′ do not (black trace).

Modeling the impact of solvated electrons on the water dynamics is challenging given the transient formation of structures only captured via rigorous quantum mechanical descriptions,^39^ in conflict with the mesoscale size of our system. For simplicity, here, we ignore chemical transformations and use instead classical MD simulations to recreate the effect of electrostatic forces stemming from injected charges. To this end, we consider a system where water and fluoride anions (F^–^) coexist in the nanochannels (SI, Section III), whereas positive charges remain in the diamond. This model is consistent with electron (rather than hole) injection into the liquid, a scenario favored here given the negative surface affinity of chemically oxidized diamond^29,30^ along with the upward band bending in NV-engineered surfaces.^40–42^

Figure 4c shows the calculated equilibrium density profiles of water and F^–^ ions for hydroxylated diamond. As in pure water, H_2_O molecules organize to form layers parallel to the solid walls. The diamond surface features regularly spaced atomic-scale pockets that can be filled by either water molecules or F^–^ ions. We find that these pockets are preferentially occupied by the ions, followed by a first monolayer of water molecules (Figure 4d). To assess the impact of these charges, we calculate the MSD of water molecules initially located in layers L1′, L2′, and L3′. As shown in Figure 4e, the MSD of first-layer water is negligible within the 6 ns span of the simulation suggesting that the molecules in this layer are effectively immobile; diffusion is faster for successive water layers, though the overall dynamics are much slower than observed in the absence of ions (we obtain similar results for alternative surface termination, SI, Section III). These findings relate to preceding MD simulations of water in the presence of externally applied electric fields, found to stabilize molecular motion to induce an ice-like structure.^43^

Proximity of nuclear spins to surface charges may potentially lead to hyperfine interactions strong enough to broaden the observed NMR spectrum, hence providing a rationale for spectral broadening, even in the presence of reduced water diffusivity (possibly the case in the bottom trace of Figure 3c). Discrete satellites in the spectrum suggest long-lived water structures, though its physical nature is presently unclear. Along the same lines, a uniform distribution of hyperfine couplings can help explain the asymmetric shape in the spectrum extracted from the ^1^H correlation signal in Figure 3b (SI, Section II).

## Discussion

Our results hint at a new, yet uncharted, dynamic regime where confinement, solvated carriers, and space charge fields combine to slow down molecular motion and possibly produce long-lived, charged molecular assemblies, likely at the solid–water interface. Additional work will be necessary to better characterize these processes, starting with the point defects serving as sources of photogenerated carriers^29,44,45^ and charge traps.^40^ Note that NV themselves could serve as local sensors to probe these fields.^46–48^

An intriguing possibility is the extension of these experiments to variable temperatures, especially below the freezing point.^49^ Structures relying on thin capping layers—where the channel height correlates with the amount of entrapped water^50^—could also prove useful to reach the limit of atomic liquid layers, where water shows a rich behavior.^51^ Additional work will also be needed to clarify the role of the confining structure in the emergence of space charge fields and whether similar dynamics can be induced in water layers adsorbed on the diamond surface. By the same token, simulations combining quantum ab initio MD, Monte Carlo, and machine learning could help implement more rigorous models without the trade-off imposed by the mesoscale size of the system.^49^ Our results may prove relevant to the implementation of biomimetic computations on aqueous electrolytic chips,^52^ the development of novel nuclear spin spectroscopy^53^ and hyper-polarization techniques,^54,55^ and the understanding of water dynamics proximal to large biomolecules.^56^

## Supplementary Material



## Data Availability

The data that support the findings of this study are available from the corresponding author upon reasonable request.

## Notes

Description of the nanochannel fabrication and experimental setup, measurement protocol and conditions; additional characterization measurements of the NV response close and far from the nanochannels; data analysis and extended results on molecular dynamics simulations (PDF)

## Notes

All source codes for data analysis and numerical modeling used in this study are available from the corresponding author upon reasonable request.
