# Supplementary material for: Slow Water in Engineered Nanochannels Revealed by Color-Center-Enabled Sensing
Source: Nano Lett. 2025 Jun 12;25(25):9960–6. doi: 10.1021/acs.nanolett.5c01344 (PMC12203581; doi:10.1021/acs.nanolett.5c01344)
Supplement: Supplementary file 1 [file nl5c01344_si_001.pdf]

Supplementary Material for

Slow water in engineered nano-channels revealed by color-center-enabled sensing

Daniela Pagliero[1], Rohma Khan[1,2], Kapila Elkaduwe[2,3], Ankit Bhardwaj[4,5], Kang Xu[1], Abraham Wolcott[1,6], Gustavo López[2,7], Boya Radha[4,5,*], Nicolas Giovambattista[2,3,*], and Carlos A. Meriles[1,2,*]

[1]*Department of Physics, CUNY–The City College of New York, New York, NY 10031, USA.*
[2]*CUNY-Graduate Center, New York, NY 10016, USA.*
[3]*Department of Physics, CUNY–Brooklyn College of the City University of New York, Brooklyn, NY, 11210, USA.*
[4]*Department of Physics and Astronomy, The University of Manchester, Manchester, UK.*
[5]*Photon Science Institute and National Graphene Institute, University of Manchester, UK.*
[6]*Department of Chemistry, San José State University, San José, CA 95192, USA.*
[7]*Department of Chemistry, CUNY–Lehman College, Bronx, NY 10468, USA.*

[*]Corresponding authors: radha.boya@manchester.ac.uk, NGiovambattista@brooklyn.cuny.edu, cmeriles@ccny.cuny.edu.

I. Materials and experimental methods

I.1 *Nanochannel fabrication*

Our nanometer-slit device comprises two hexagonal boron nitride (hBN) flakes, prepared by mechanical exfoliation from bulk, high-purity hBN[1]. We first exfoliate a 5.6 nm thick layer and transfer it to a silicon wafer featuring a 90 nm layer of SiO_2; we subsequently resort to electron beam lithography (Zeiss SmartSEM + Raith ELPHY Quantum NNANOSUITE V6.0) and dry etching (Plasmalab 100 ICP-65, Oxford) to produce a two-dimensional (2D) striped array, as shown in Fig. 1 of the main text. Collectively, the hBN strips — each approximately 275 nm wide, separated from nearest neighbors by 185 nm — function as spacers defining the channels height[2]. To cap the array, we transfer a 70-nm-thick mechanically-exfoliated hBN flake to another SiO_2 substrate; this thickness provides sufficient mechanical rigidity to prevent sagging of the slits in air or swelling in water. After each transfer, we bake the device on a hotplate at 170°C for 30 minutes to remove any absorbates and provide good adhesion; this includes the transfer of the top-spacer stack to its final diamond substrate, which we carry out via adhesion to poly-methyl-methacrylate (PMMA). Once on the diamond, we use electron beam lithography and dry etching to open two windows on either end of the spacers, hence allowing H_2O molecules to access the channels upon exposure to water vapor. Prior to magnetic resonance measurements, we use an optical microscope (Nikon Eclipse LV100ND) to observe the hBN flakes after exfoliation and for device inspection at all stages. We also employed atomic force microscopy (Dimension Fastscan with Bruker's Scanasyst) to probe the height profile of the hBN spacers. We summarize the fabrication process in Fig. S1.

I.2 *Operating geometry and channel filling protocol*

The diamond crystal sits within the main chamber of a 3D printed microfluidic cell sealed with transparent windows on both sides to allow for optical measurements (Fig. S2). A through channel and two side ports connect the chamber to the outside, hence allowing for a controlled environment. A double, 0.5-mm-diameter loop of thin (44 gauge) coated copper wire glued to one of the windows serves as the MW antenna.

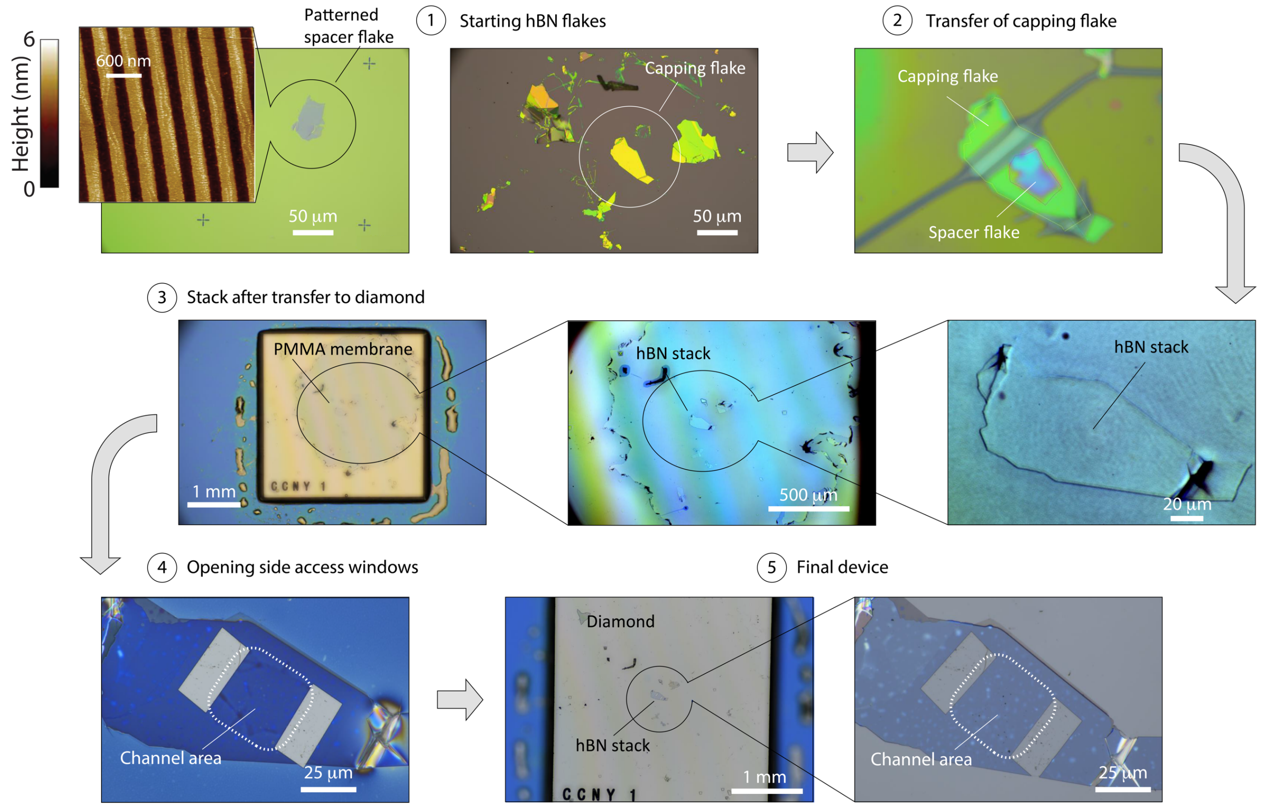

Figure S1 | Nanochannel fabrication. Starting with hBN flakes on separate SiO_2 substrates, we first use electron beam lithography to produce a striped, 5.6-nm-tall spacer pattern, which we then cap with a 70-nm-thick flake for upper channel confinement (steps ① and ②, the dark diagonal shadow in ② is a PMMA membrane holding the top flake). We then use PMMA to transfer the stack into diamond (step ③), where we open two side windows via electron beam lithography and dry etching (step ④). The last two images show the end structure after PMMA removal (step ⑤).

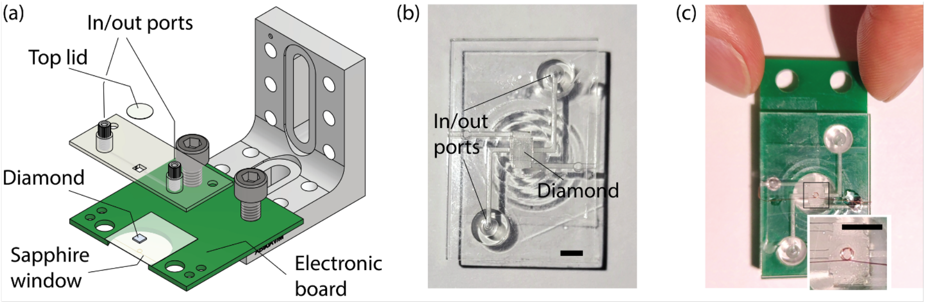

Figure S2 | Controlling the nanochannel environment. (a) Schematics of the sample mount including the microfluidics chip and antenna board. (b) Image of the microfluidic chip. (c) Photograph of the final device. The insert is a zoomed-out image of the squared section. The scale bar in (b) and (c) is 2 mm.

To water-fill the channels, we first flush MilliQ H_2O through the ports to flood the sample chamber, a wetting step lasting for about an hour. We then flow argon gas — previously humidified via bubbling in MilliQ water within a separate container — through the chip's inlet port and into the sample chamber. This ~12-hour-long step removes bulk water from the chamber while maintaining a humid atmosphere. No gas flow takes place during measurements.

I.3 *Experimental setup*

We use a home-built confocal microscope with a 532 nm laser and an acousto-optic modulator (AOM) to control laser pulsing with time precision down to 10 ns. Sample illumination and photon collection rely on a 0.9-numerical-aperture Olympus objective with 100× magnification; the laser power reaching the NVs is typically 3 mW. We use a galvo mirror system for XY scans and beam positioning; we achieve Z control with a piezoelectric stage. A white light source steered into the same objective allows us to alternatively obtain optical images, which we use to ensure correct positioning of the laser beam on the channel area. We detect the NV fluorescence via a 25-µm-fiber-coupled avalanche-photo-detector (APD-Excelitas) after a dichroic mirror and a long-pass filter; we use a SpinCore PulseBlaster to gate the APD and the AOM. To manipulate the NV spin, we apply pulsed microwave (MW) excitation from a signal generator (Rohde & Schwarz). We feed the I and Q channels from an arbitrary wavefunction generator (AWG-SE5082 from Tabor electronics) into an IQ mixer (ADL5375, 400 MHz to 6 GHz Broadband Quadrature Modulator), which allows us to attain MW phase control. Sample MW excitation rely on an amplifier (Minicircuits ZHL-25W-272+) and an on-chip, 50-Ohm-terminated antenna (see above). We use a permanent magnet to create a magnetic field of ~40 mT along a direction forming an angle of 54.6 deg. relative to the surface normal and parallel to one of the NV crystallographic axes. All experiments take place under ambient conditions.

I.4 *NV spin characterization and control*

We use a 2×2×0.5 mm^3, [100] electronic grade diamond crystal from E6, subsequently processed by QuantumDiamond. The crystal was overgrown with a ^{12}C-enriched (99.99%), 1-µm-thick layer and then polished to bring down the average surface roughness to less than 1 nm. ^{15}N-implantion (1×10^{12} cm^{-2}, 3 keV) followed by thermal annealing (850 ^0C) resulted in an 8-nm-deep NV layer with approximate concentration of 10^2 centers per μm^2. Prior to fabricating the nanochannel structure, we followed standard acid boiling protocols for chemical surface oxygenation; we limit subsequent surface cleanings to acetone immersion so as to preserve the device integrity. Standard PL imaging of the device in the channel area shows exposure to water — both in liquid and vapor forms — has a significant impact on the system brightness. In particular, we find the NV ensemble fluorescence changes after channel filling to gradually become dimmer, the state during measurements (Fig. S3a). These changes are largely reproducible and hence suggest charge state conversion processes with efficiency dictated by local heterogeneities in defect concentration and/or surface termination. We warn, however, there is some residual variability in the PL images we obtain in any given water-exposure-and-measurement cycle, implying it is challenging to ensure we examine the exact same (diffraction-limited) sites in subsequent experimental rounds (see below). All experiments are interlaced with a site tracking protocol that minimizes axial and lateral drifts over long-duration measurements.

We use MW tuned to the $|m_S = 0\rangle \leftrightarrow |m_S = -1\rangle$ transition (see Fig. S3b for a schematic energy diagram). Note that in the limit of alignment between the applied magnetic field and the chosen NV crystallographic axis, optical illumination leads to ^{15}N spin polarization, thus resulting in a narrower transition (Fig. 2b in the main text). With the exception of pulsed ODMR measurements, however, we make use of short pulses (typically, ~70 ns, Fig. S3c) for broadest spectral excitation, hence rendering ^{15}N spin pumping convenient but unnecessary. We attain ~4% spin contrast during NV optical readout (see Rabi amplitudes in Fig. S3c), and measure characteristic Hahn-echo (dynamical decoupling) coherence times reaching 4.5 µs (19.8 µs), typical for shallow NVs at the present concentration[3] (Figs. S3d and S3e, respectively).

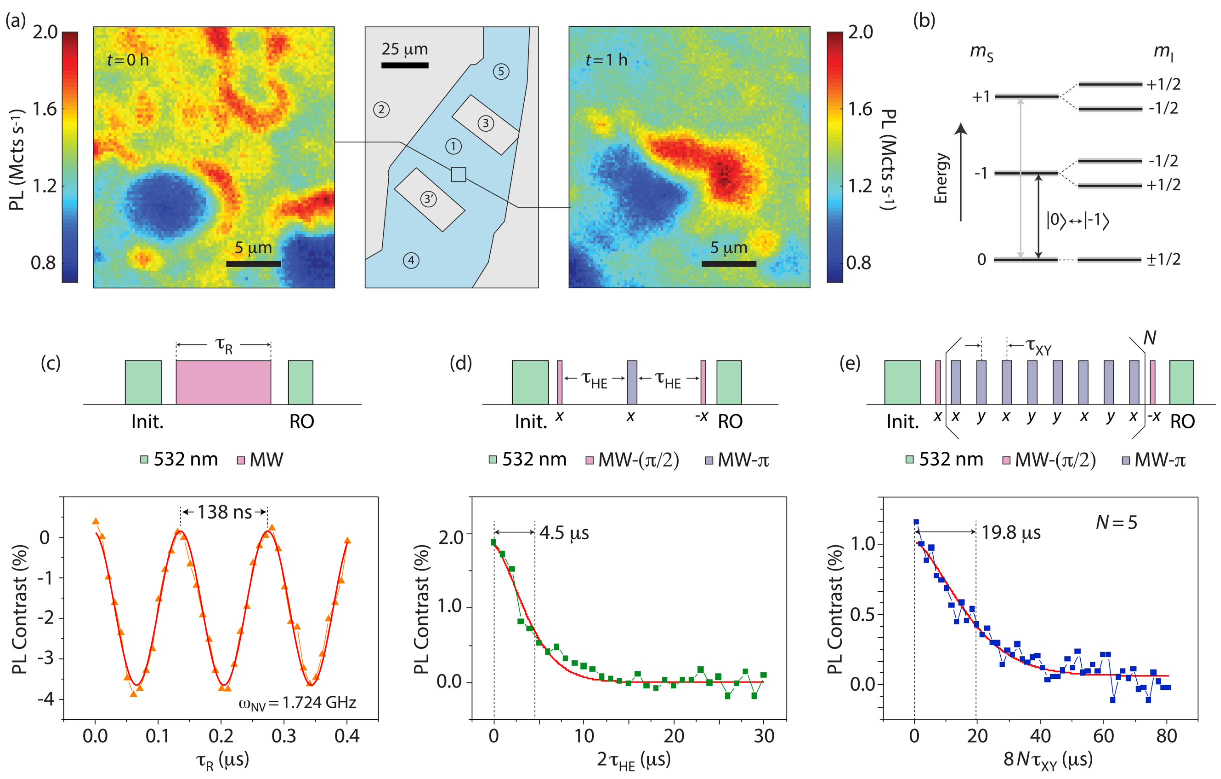

Figure S3 | NV characterization. (a) NV fluorescence imaging under green excitation of a region in the channel area immediately after exposure to liquid water and humidified argon (left) and an hour later (right). Significant changes in the PL brightness suggest a modification of the NV charge state. (b) Simplified energy diagram for the NV spin in the ground state orbital assuming a magnetic field B aligned with the NV axis. Throughout our experiment, we tune the MW to the $|0\rangle \leftrightarrow |-1\rangle$ transition at the applied B; note that hyperfine coupling with the host ^{15}N — a spin-1/2 nucleus — leads to splitting of the ODMR dip (Fig. 2b in the main text). (c through e) Characteristic NV response to Rabi, Hahn-echo, and XY8-N protocols. In all cases, the solid red trace indicates a fit in the form of a damped sinusoidal (Rabi measurements) or a stretched exponential (Hahn-echo and XY8-5). In all protocols, the typical readout (RO) and initialization (Init.) times are 0.75 and 10 μs, respectively; the 532-nm laser intensity is 1.5 mW in all pulsed experiments.

I.5 *NV-detected ^1H magnetic resonance*

We implement ^1H nuclear magnetic resonance (NMR) sensing with the help of XY8-N dynamical decoupling protocols, selectively sensitive to AC magnetic fields whose frequencies match twice the time τ_{XY} separating consecutive pulses in the train[4]; an NMR spectrum emerges as one records the NV spin signal for varying τ_{XY} in the vicinity of the ^1H Larmor frequency, ω_n, at the applied magnetic field (typically in the range 38–45 mT). Correlation measurements use the same nuclear-spin-noise-sensing principle[5,6], except that this time it is the interval $\tilde{\tau}$ separating the encoding and decoding dynamical decoupling blocks that varies in subsequent runs; the end result is the equivalent of a "free induction decay" at the frequency defined by τ_{XY}.

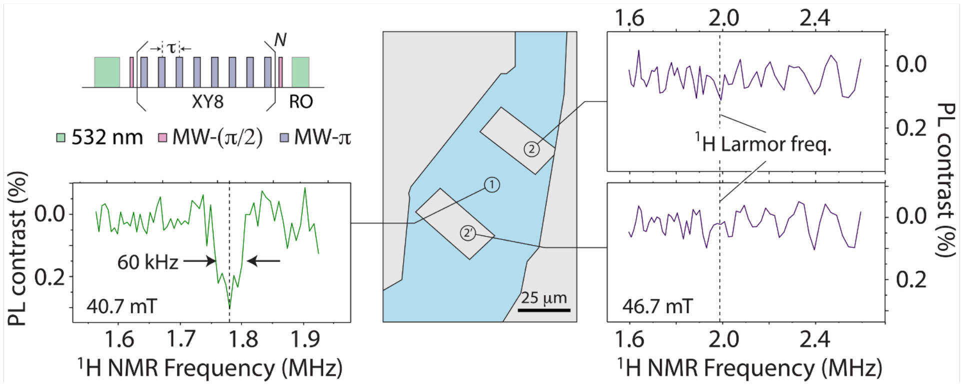

Figure S4 | NV-NMR in and out of the channel area. (Left) Pulse sequence and NV-NMR signal from a site in the channel area, Section ① (top and bottom, respectively). (Center) Schematic of the patterned hBN flake; ① and ② respectively denote the channel and window areas. (Right) We observe no ^1H NV-NMR signal from either window in the structures (sections ②).

We divide our experiments into “measurement rounds”, each starting with nanochannel exposure to water followed by extended observation periods, whose duration (typically two to three weeks) depends on the rate of water evaporation (noticeable despite chip encapsulation of the diamond crystal, Fig. S2). Figure 3a in the main text illustrates this point with the ^{1}H NMR spectra observed immediately before and after a channel fill (marking the separation between two consecutive measurement cycles). To compensate for long-term system instabilities — created, e.g., by lab temperature fluctuations — we limit individual NMR data collection runs to 12–15 hour windows, which we intersperse with ODMR and Rabi measurements for regular MW frequency and amplitude monitoring; we also integrate an automated “site tracking” protocol to avoid laser beam drifting during prolonged experiments. For future reference, pulsed green optical excitation — with characteristic intensity of order 6 GW/m^2 in our diffraction-limited microscope — is invariably present throughout all these protocols for optimal SNR.

We limit our discussion to signals recorded by NVs within the channel area. Although water is expected to adsorb onto all diamond surfaces during the channel fill, we failed to observe ^{1}H NMR signatures outside this section of the structure (Fig. S4). This could indicate the adsorbed water layer is just too thin under normal operating conditions and falls below our detection limit, though it could also be a consequence of the NMR resonance being too broad; this later case, in turn, does not necessarily imply high water diffusivity, as the broadening could originate from hyperfine interactions with surface charge (see below, Section II).

II. Data analysis

II.1 *Self-diffusivity of water under nano-channel confinement*

To characterize water diffusion, we implement NV correlation spectroscopy using two consecutive XY8-*N* pulse trains resonant with the ^{1}H Larmor frequency. Reproduced in Fig. S5a, this protocol probes the auto-correlation function of the NV time evolution in the presence of the statistical magnetic field created by the sample nuclear spins. The stochastic nature of this nuclear “spin noise” makes the scheme sensitive to diffusion because any possible correlation between the phases picked up by a probe NV during each encoding segment vanishes if the source nuclear spins diffuse away from the sensing volume during the interval $\tilde{\tau}$ separating the pulse trains. The correlation signal takes therefore the form[5]

$$C(\tilde{\tau}) \propto \langle B_{\mathrm{n}}^2 \rangle \cos(\omega_{\mathrm{n}} \tilde{\tau})\, F_{\mathrm{PL}}\left(\frac{\tilde{\tau}}{T_{\mathrm{D}}}\right), \tag{1}$$

where $\langle B_{\mathrm{n}}^2 \rangle$ is the root-mean-square field produced by the nuclear spins, ω_{n} is the nuclear Larmor frequency, and $F_{\mathrm{PL}}\left(\frac{\tilde{\tau}}{T_{\mathrm{D}}}\right)$ is a decay envelope, where T_{D} denotes the molecular self-diffusion time. The latter is connected to the sample self-diffusion coefficient D via the relation $d \approx \sqrt{6DT_{\mathrm{D}}}$, with d representing the detection volume radius (in turn, approximately equal to the NV distance to the surface[7]).

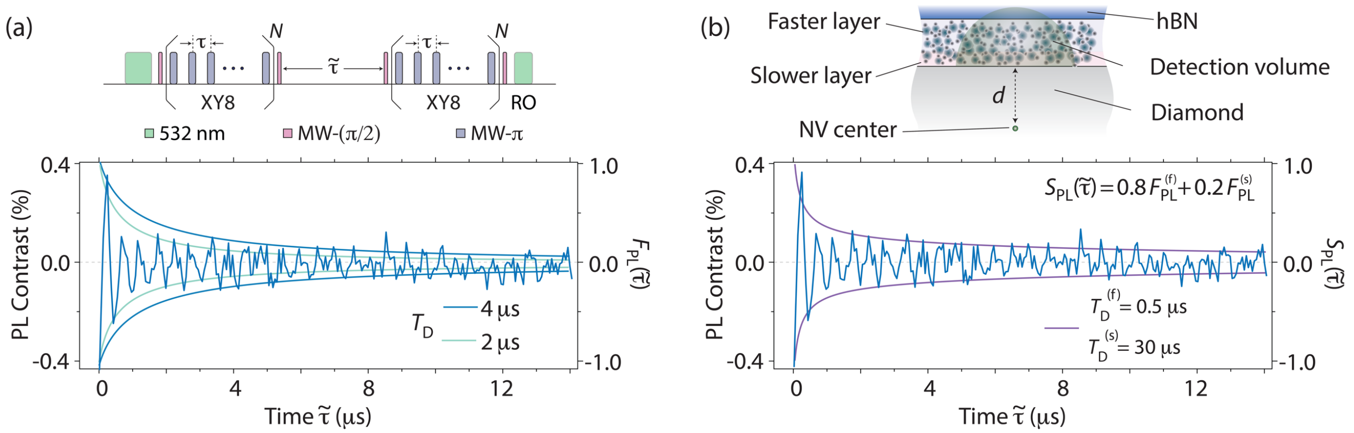

Figure S5 | ^{1}H correlation spectroscopy as a probe of water diffusion. (a) Correlation protocol and ^{1}H correlation signal (reproduced from Fig. 3b in the main text). The blue (green) envelope was calculated from Eq. (2) assuming $T_{\mathrm{D}} = 4$ μs ($T_{\mathrm{D}} = 2$ μs). Agreement is poor at short times when $T_{\mathrm{D}} \gtrsim 4$ μs; we observe the converse if $T_{\mathrm{D}} \lesssim 2$ μs. (b) (Top) We assume a two-layer model where water molecules proximal to the diamond surface move more slowly. (Bottom) Same as in (a) but assuming an envelope of the form $S_{\mathrm{PL}}(\tilde{\tau}) = 0.8F_{\mathrm{PL}}^{(\mathrm{f})}(\tilde{\tau}) + 0.2F_{\mathrm{PL}}^{(\mathrm{s})}(\tilde{\tau})$ where $F_{\mathrm{PL}}^{(\mathrm{f})}$ ($F_{\mathrm{PL}}^{(\mathrm{s})}$) has characteristic time $T_{\mathrm{D}}^{(\mathrm{f})}$ ($T_{\mathrm{D}}^{(\mathrm{s})}$). We attain reasonable agreement both after short and long evolution intervals.

The decay envelope $F\left(\frac{\tilde{\tau}}{T_{\mathrm{D}}}\right)$ has been often assumed to follow an exponential dependence with characteristic decay time T_{D}[6]. A more detailed analysis that takes into consideration the sensing geometry[8] — restricted by the host diamond to half a sphere — shows instead the response at long times $\tilde{\tau} \gg T_{\mathrm{D}}$ has the form $F_{\mathrm{PL}}\left(\frac{\tilde{\tau}}{T_{\mathrm{D}}}\right) \propto \left(\frac{\tilde{\tau}}{T_{\mathrm{D}}}\right)^{-\frac{3}{2}}$, where the label in F stands for the predicted power law dependence. Compared to an exponential function, the slower power law decay better captures the long-lived shape of the nuclear spin correlation signals derived from observations of ^{1}H spins in oil drops on the diamond surface[6,8].

The bottom half in Fig. S5a compares the present results with the predicted correlation envelope F_{PL}, whose complete functional dependence[8] we reproduce below for completeness, namely

$$F_{\mathrm{PL}}(z) = \frac{4}{\sqrt{\pi}}\left(z^{-\frac{3}{2}} - \frac{3}{2}z^{-\frac{1}{2}} + \frac{\sqrt{\pi}}{4} + 3z^{\frac{1}{2}} - \frac{3\sqrt{\pi}}{2}z + \sqrt{\frac{\pi}{z}}\,\mathrm{erfc}\left(z^{-\frac{1}{2}}\right)\exp\left(z^{-1}\right)\left(-z^{-\frac{3}{2}} + z^{-\frac{1}{2}} - \frac{7}{4}z^{\frac{1}{2}} + \frac{3}{2}z^{\frac{3}{2}}\right)\right) \quad (2)$$

where $z \equiv \tilde{\tau}/T_{\mathrm{D}}$. Indeed, the measured correlation signal does show a non-exponential envelope characterized by a quick decay at early times followed by a longer-lived coherence, but we find only qualitative agreement with Eq. (2) suggesting the diffusion dynamics of entrapped water is more complex. Super-imposed solid traces corresponding to Eq. (2) for different diffusion times — specifically, 2 and 4 μs — show longer values for T_{D} better describe the response at long times but fail to capture the rapid decay seen for short evolution intervals. This behavior could stem from heterogeneity in the self-diffusion coefficient, longer for water layers closer to the surface. For example, we attain improved agreement with a simplified, two-layer model where $S_{\mathrm{PL}}(\tilde{\tau}) = \alpha F_{\mathrm{PL}}^{(\mathrm{f})}(\tilde{\tau}) + \beta F_{\mathrm{PL}}^{(\mathrm{s})}(\tilde{\tau})$ with characteristic diffusion times $T_{\mathrm{D}}^{(\mathrm{f})} = 0.5$ μs and $T_{\mathrm{D}}^{(\mathrm{s})} = 30$ μs and normalized weights $\alpha = 0.8$, $\beta = 0.2$ (Fig. S5b). Using $d = 8$ nm as the characteristic size of the detection volume, we derive self-diffusion constants $D^{(\mathrm{f})} = d^2/6T_{\mathrm{D}}^{(\mathrm{f})} \sim 21 \times 10^{-12}$ m^2 s^{-1} and $D^{(\mathrm{s})} \sim 0.4 \times 10^{-12}$ m^2 s^{-1}, much below the self-diffusion coefficient of bulk water (2.3×10^{-9} m^2 s^{-1}). A similar analysis of the correlation signal in Fig. 4b of the main text — not shown here for brevity — indicates the relaxation time in this slower layer can exceed 100 μs, hence leading to $D \leq 0.1 \times 10^{-12}$ m^2 s^{-1}. Our analysis is qualitatively consistent with the heterogeneity observed for entrapped water by MD modeling (see below, Section III) where molecular diffusion is found to depend sensitively on the distance to the walls when charges are present (see also Figs. 4c through 4e in the main text).

II.2 *Hyperfine interactions*

Inspection of the correlation signal's Fourier transform shows an asymmetric spectrum with maximum at the anticipated ^{1}H Larmor frequency — 1.91 MHz at the applied magnetic field — but surrounded by shoulders spanning a ~2 MHz range (Fig. S6a). Since this broadening largely exceeds that possible via ^{1}H chemical shifts or internuclear dipolar couplings, we theorize the observed pattern stems from a different spin interaction, possibly hyperfine in

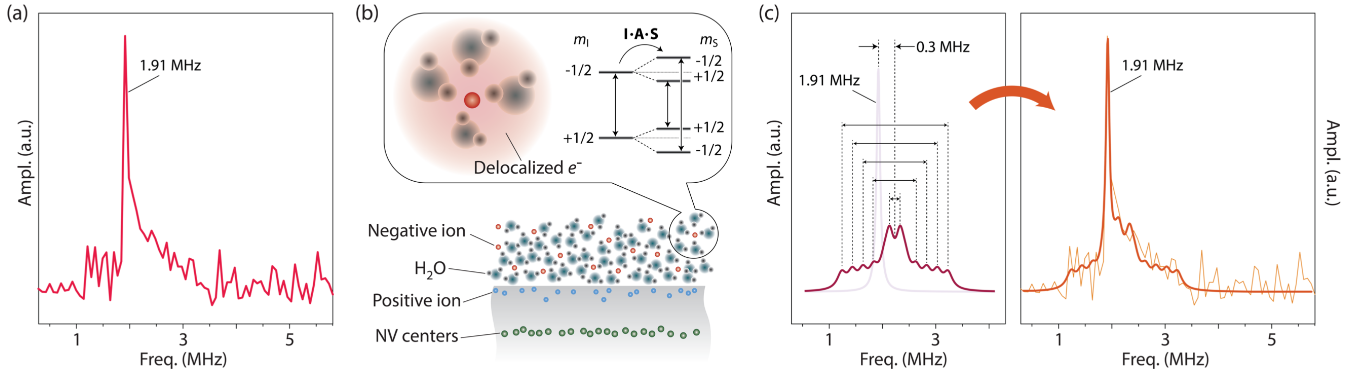

Figure S6 | Hyperfine-induced broadening? (a) Fourier transform of the ^{1}H spin correlation signal as shown in Fig. S5. Besides the main nuclear spin resonance at the ^{1}H Larmor frequency, the spectrum displays an asymmetric broadening spanning ~2 MHz. (b) Photogenerated carriers lead to the formation of an electric double layer, with positive charge likely accumulating at the interface and electrons solvating into the fluid. We hypothesize hyperfine couplings between near-surface ^{1}H nuclei and the electronic spins of these carriers — respectively denoted by vector operators **I** and **S** — lead to splitting of the proton NMR resonance with amplitude $||\mathbf{A}||$. Arrows on the energy diagram (right hand side insert) indicate allowed transition frequencies. (c) We describe the ^{1}H NMR spectrum as a superposition of contributions from water structures experiencing different hyperfine couplings (here discretized to take five different values). The combination of hyperfine-shifted and hyperfine-free contributions lead to a pattern consistent with experiment. Note the positive ~0.3 MHz shift of the hyperfine pattern center frequency, suggestive of strong non-diagonal contributions to the hyperfine tensor.

nature. Supporting this hypothesis is the recent observation of charge injection from shallow NVs and coexisting surface traps under green excitation[9,10], here activated by the combination of day-long measurement times and the insulating nature of all components in the nanochannel structure (deprived from electrical grounding and hence prone to the formation of space charge fields). The injection of solvated electrons, not holes, seems the most likely scenario given the negative electron affinity of diamond surfaces in contact with water[11] (Fig. S6b) along with the upward band bending characteristic of NV-engineered surfaces[12-15].

To model the interaction between ^{1}H spins and the excess electron in a given molecular cluster, we write the Hamiltonian as

$$H = -\omega_n I_z + A I_z S_z + A' I_x S_z \,. \tag{1}$$

In the above expression, ω_n is the proton Larmor frequency at the applied magnetic field, **I** and **S** are vector operators respectively representing the ^{1}H and electron spins, A and A' denote hyperfine coupling constants, and we assume $\hbar = 1$. In the representation corresponding to the eigenstates of I_z and S_z, the Hamiltonian takes a block-diagonal form; for the $|m_S = -1/2\rangle$ manifold, the eigenenergies are given by

$$E_{\pm}^{(-1/2)} = \pm\sqrt{\frac{1}{4}\left(\omega_n + \frac{A}{2}\right)^2 + \frac{A'^2}{16}} \approx \pm\frac{1}{2}\left(\omega_n + \frac{A}{2} + \frac{A'^2}{8\omega_n}\right), \tag{2}$$

where we assume $A \cong A' < \omega_n$ (see energy diagram in Fig. S6b). Similarly, we find for the $|m_S = +1/2\rangle$ manifold

$$E_{\pm}^{(+1/2)} = \pm\sqrt{\frac{1}{4}\left(\omega_n - \frac{A}{2}\right)^2 + \frac{A'^2}{16}} \approx \pm\frac{1}{2}\left(\omega_n - \frac{A}{2} + \frac{A'^2}{8\omega_n}\right). \tag{3}$$

Since the NMR-allowed transitions must preserve the electron spin projection, we find the resonance frequencies in the hyperfine doublet take the values

$$\omega_n^{\pm} = \omega_n \pm \frac{A}{2} + \frac{A'^2}{8\omega_n}\,. \tag{4}$$

Equation (4) implies that in the presence of hyperfine couplings, the ^{1}H resonance splits into two peaks, each separated from the center frequency by $A/2$. Note that the average frequency $(\omega_n^+ + \omega_n^-)/2$ shifts from ω_n by a positive amount $A'^2/8\omega_n$; as an example, for the present magnetic field conditions (ω_n~2 MHz) and assuming $A' \approx 1$ MHz, we obtain $A'^2/8\omega_n \approx 60$ kHz.

Now, we can tentatively rationalize the ^{1}H spectrum as the result of heterogeneous contributions from protons experiencing hyperfine couplings varying over a ~2 MHz range, each group manifesting as a spectral doublet (whose center is potentially blue-shifted from the ^{1}H Larmor frequency). To prove this point, we choose five different hyperfine couplings with amplitudes A_i, $i = 1 \cdots 5$; in all cases we use A_i'~2 MHz, impose a linewidth of 0.1 MHz, and select varying amplitudes to create the hyperfine pattern shown on the left panel of Fig. S6c. Assuming the inner water layers experience negligible hyperfine splitting, we can qualitatively reproduce the observed spectrum if we supplement the hyperfine shifted pattern with a resonance peak at the ^{1}H Larmor frequency (right-hand side panel in Fig. S6c). Note that the same rationale can be extended to reproduce the bottom spectrum in the series of Fig. 3c of the main text, except that in this case, the observation of resolved satellites suggests water molecules and carriers organize to adopt preferred structures featuring specific hyperfine coupling constants.

Notably, the hyperfine strengths we derive have order of magnitude comparable to that extracted for protons in the solvation layer of ions dissolved in bulk water as measured via inductive liquid-state NMR[16]. We warn, however, the above assignments should only be seen as a crude guide since the varying relative amplitudes of the resonance pairs and limited signal-to-noise ratio (SNR) combine to create some ambiguity (a complication that could be mitigated by resorting to improved readout protocols[17]). More importantly, while H_2O molecules are known to coordinate around solvated electrons, the resulting clusters diffuse rapidly[18] and are thus difficult to reconcile with our observations without also imposing temporal stability over a time scale comparable to the duration of our measurements. Lastly, we emphasize different measurement runs — understood as the set of experiments during the time interval separating two consecutive channels fills, typically 2 to 3 weeks — do not necessarily yield the exact same evolution in the ^{1}H NMR spectra we presented in Fig. 4c of the main text, perhaps a consequence of the varying nature of the space charge fields forming under laser excitation. This variability could also originate from heterogeneities in the channel structure and/or surface termination, as residual NV PL changes between different rounds complicate our ability to replicate the same

(diffraction-limited) location for measurements belonging to different runs.

We note that experiments at higher magnetic field (e.g., 500 mT and greater) could provide additional information on these off-diagonal hyperfine terms. However, reaching this regime poses substantial experimental challenges. Our system relies on permanent magnets, which are limited to ~100 mT, and higher fields introduce stronger gradients that destabilize NV resonance conditions over the long acquisition times (~24 h) required here. Additionally, spin manipulation at higher fields demands microwave hardware and infrastructure beyond our current capabilities.

III. Molecular dynamics simulations

We perform classical molecular dynamics (MD) simulations of a system composed of $N{\sim}30000$ water molecules, with and without additional charges, sandwiched by an hBN monolayer and a diamond crystal as described in the main text. To account for the chemical heterogeneity resulting from wet oxidation, we consider four diamond surfaces terminated with either (i) H, (ii) O, (iii) OH, and (iv) a combination of H and OH groups (arranged periodically over the surface). Water molecules are represented using the TIP4P/2005 water model[19]. During the MD simulations, the hBN and diamond atoms are immobile except for the H atoms belonging to the diamond OH groups (cases (iii) and (iv)). The distance between the hBN monolayer and the outmost C atoms of diamond is set to ~5.771-5.856 nm depending on the diamond surface considered. The hBN/diamond surfaces are perpendicular to the z-axis and have an area $A = \Delta y \cdot \Delta x$ with $\Delta y = 13.137 - 13.197$ nm and $\Delta x = 13.137 - 13.197$ nm depending on the surface considered (see below). The diamond crystal thickness is $\Delta z = 1.151 - 1.226$ nm (depending on the diamond surface termination). Increasing the values of Δz does not affect our results because the additional C atoms are located at least 1.15 nm apart from any water molecule in the confined volume and the corresponding C-water interactions are null (the Lennard-Jones interactions between the water molecules and the diamond C atoms are short-ranged, and are evaluated within a cutoff distance $r_c = 1.10$ nm). All MD simulations are performed at constant temperature ($T = 300$ K) and volume. The system box side lengths are ($L_z = 30.000, L_y = \Delta y, L_x = \Delta x$) and periodic boundary conditions apply to all three directions (note that L_z is much larger than the separation between the plates (<6 nm) and the diamond thickness Δz; this extra empty space is left to minimize any potential effect that may be due to the periodic boundary conditions along the z-direction).

All computer simulations are performed using the GROMACS software package (2022.6-spack)[20]. The system temperature is maintained using a Nose-Hoover thermostat with a time constant of 0.5 ps[21,22]. Coulombic interactions are evaluated using the particle mesh Ewald technique[23] with a Fourier spacing of 0.12 nm. For both the Lennard-Jones (LJ) and the real space Coulomb interactions, we use a cutoff of $r_c = 1.10$ nm. To control the water OH covalent bonds lengths, we use the LINCS (Linear Constraint Solver) algorithm. MD simulations are performed for 8 ns which is long compared with the relaxation time of bulk TIP4P/2005 water (τ~50–100 ps) at $T = 300$ K and $P = 0.1$ MPa[24] (under these conditions, it takes <100 ps for the mean-square displacement of the water molecules to be > 1 nm^2). The simulation time step is $dt = 1$ fs.

III.1 *Systems preparation*

For each diamond surface studied, we carry out three kinds of MD simulations as follows:

A) We first perform MD simulations of a thick film of water (~6.5 nm-thick; $N = 33628$) covering the whole diamond surface (Fig. S7a). The system is simulated for 4 ns allowing for water to reach equilibrium. After a few ps, water molecules at the diamond interface rearrange into three layers (red line in Fig. S7c). These water layers are separated from the liquid-vapor interface (at $z > 5.5$ nm) by a ~4 nm-thick volume of bulk-like water. We note that this bulk-like water is at $P{\sim}0$ MPa since it is in contact with a vapor volume and the average liquid vapor interface is flat. Accordingly, the density of the thick bulk-like water volume is independent of the surface considered; in all cases we find that the density of the bulk-like water is close to $\rho_{\text{bulk}} \sim 0.997$ g/cm^3 which is the density of bulk TIP4P/2005 water at $P = 0.1$ MPa and $T = 300$ K[19] (see Fig. S7c).

B) A second family of MD simulations is then carried out by adding an hBN monolayer at a distance $d = 5.771 - 5.856$ from the diamond surface (depending on the diamond surface termination, Fig. S7b). The distance d is selected so the density of the confined water in the intermediate region between the hBN/diamond surfaces matches the density of bulk (TIP4P/2005) water, $\rho_{\text{bulk}} \sim 0.997$ g/cm^3 (see Fig. S7c).

C) In order to include negative charges in the confined water volume, we proceed as follows. For a target diamond surface, we take an equilibrated configuration of the corresponding diamond-water MD simulation described in (*B*). Then, we replace randomly N_q water molecules by fluoride anions. The fluoride anions are modeled using the Madrid2019 force field[25] where each anion has an effective charge $q = -0.85\ e$. Hence, to make the system charge-neutral, we also add a total positive charge $N_q|q|$ to the diamond crystal. This charge is distributed evenly over the C

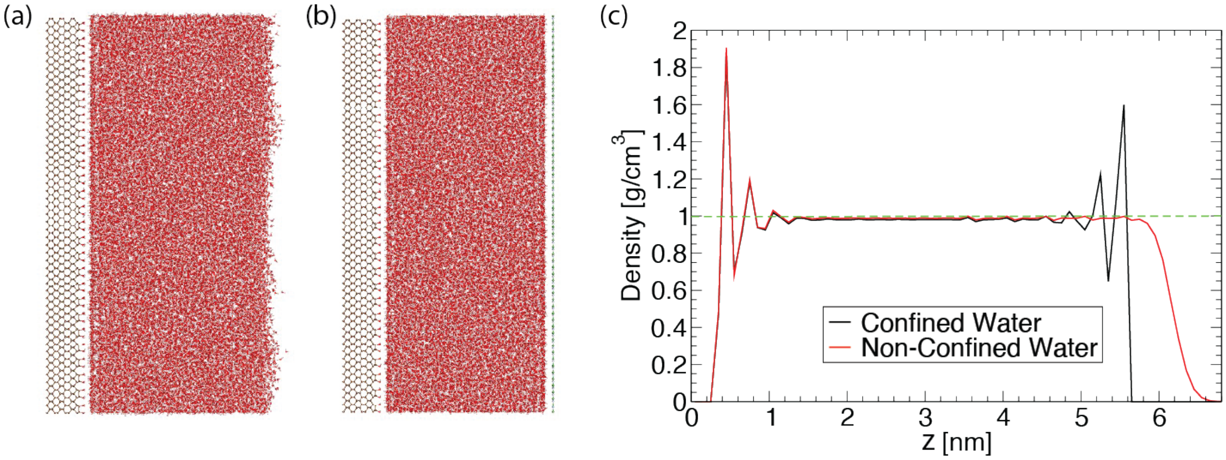

Fig. S7 | System preparation. (a) Snapshot from MD simulations of a ~6.5 nm-thick water film on a diamond crystal passivated with OH groups. The water film is separated from the vapor phase by a flat interface that fluctuates during the MD simulation. (b) Snapshot from MD simulations of the system shown in (a) after adding a hBN monolayer to confine the water volume. (c) Density profile of water as a function of the distance z from the diamond surface for the systems shown in (a) and (b). In both cases, three water layers form near the diamond interface, and a bulk-like volume of water exists at approximately $z > 1.5$ nm. In system (a), red line, the density profile decays smoothly to zero as the vapor phase is reached; in system (b), three water layers from next to hBN. The diamond-hBN separation in system (b) is tuned to match the density of bulk TIP4P/2005 water at $z{\sim}2-4$ nm (the horizontal dashed line in (c) indicates the density of bulk TIP4P/2005 water at $P = 0.1$ MPa and $T = 300$ K, $\rho_{\mathrm{bulk}} \sim 0.997$ g/cm^3).

monolayer of the diamond crystal that is located closest to the confined water (these C atoms form a covalent bond with the surface O and are shown in blue in Fig. 4d of the main text). This C monolayer contains N_c atoms ($N_c = N_q = 2704$ in the case of diamond crystals passivated with H, OH, and both H/OH groups; $N_c = 2738$ for the diamond terminated with O groups). We note that replacing the water molecules by fluoride leads to a redistribution of water molecules and to a decrease in the overall density of water in the confined volume. We will later see this can lead to the formation of vapor-like domains (see below Fig. S13a). We note that reducing the separation between the hBN monolayer and diamond suppresses the formation of vapor domains while leaving our results unaffected.

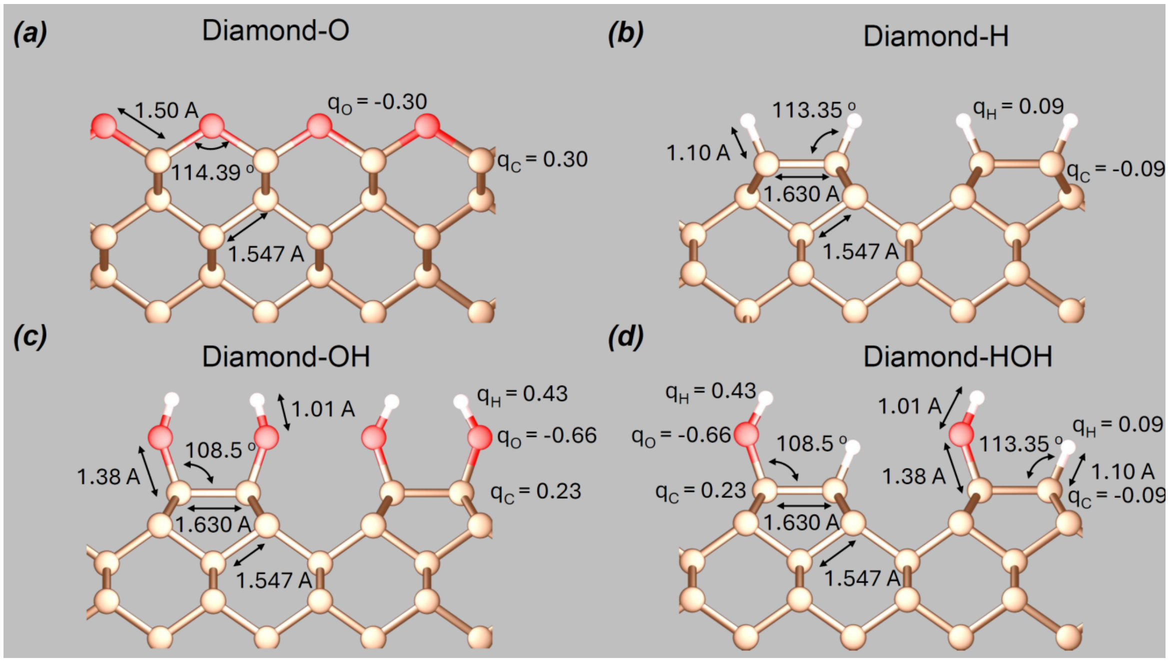

Fig. S8 | Structure of the diamond surfaces. Termination with (a) O, (b) H, (c) OH, and (d) both H and OH groups. The bond lengths, angles, and partial charges are indicated; LJ interaction parameters are given in Tables 1-4. The partial charges of the O and H atoms are adapted from Ref. [26]; the partial charges of the C atoms forming a covalent bond with the surface O and H atoms are such that the total charge of the C-H and C-O-H groups are zero.

III.2 ***Model surfaces for diamond and hBN***

Diamond. Figure S8 shows side views of the four diamond surfaces considered. The C100 surface of diamond is exposed to the water molecules, and it is passivated with either (a) O, (b) H, (c) OH, or (d) H and OH groups. The diamond atoms are maintained at fixed positions except for the H atoms of the hydroxyl groups (cases (c) and (d)). These H atoms are mobile, but they are constrained by (i) an OH stretching bond potential of the form

$$V_{\mathrm{OH}}(r) = \tfrac{1}{2}\, k_{\mathrm{r}}\, (r - r_0)^2, \tag{5}$$

where r is the OH distance, and (ii) by an HOC angle potential given by

$$V_{\mathrm{HOC}}(\theta) = \tfrac{1}{2}\, k_\theta\, (\theta - \theta_0)^2. \tag{6}$$

Parameters k_r, k_θ, r_0, and θ_0 are adapted from the CHARMM force field for alcohols and carbohydrates[26], specifically, $k_r = 456056.00$ kJ/mol/nm, $k_\theta = 481.160$ kJ/mol/rad^2, $r_0 = 0.09600$ nm and $\theta_0 = 106.00^{\circ}$. The structure of the diamond crystals terminated with H, O, and OH groups (Figs. S8a-c) are adapted from Refs. [27, 28]; the structure of the diamond crystal passivated with both H and OH groups (Fig. S8d) is a model surface that we created based on the diamond surfaces terminated with H and OH groups (Figs. S8b and S8c). The covalent bond lengths and bond angles among the immobile atoms are indicated in the figures. Only the H and O atoms at the surface, and the C atoms forming a covalent bond with the O and H atoms have a non-zero partial charge; the corresponding partial charges are also indicated in the figures. The LJ parameters ε and σ for the atoms in the different walls are given in Tables S1-S4. The Lorentz-Berthelot combination rules are used to generate the LJ parameters among the diamond atoms and the anions/water atoms.

Table S1. LJ Params for the diamond crystal terminated with O atoms.

**Atom type**	**σ [nm]**	**ε [kJ/mol]**
**O (in C-O)** [a]	0.293997	0.50208
**C (in C-O)** [b]	0.321400	0.23633
**C** [b]	0.321400	0.23633

[a] Adapted from Ref. [26]; [b] From Ref. [29].

Table S2. LJ Params for the diamond crystal terminated with H atoms.

**Atom type**	**σ [nm]**	**ε [kJ/mol]**
**H (in C-H)** [a]	0.235200	0.09205
**C (in C-H)** [b]	0.321400	0.23633
**C** [b]	0.321400	0.23633

[a] Adapted from Ref. [26]; [b] From Ref. [29].

Table S3. LJ Params for the diamond crystal terminated with OH groups.

**Atom type**	**σ [nm]**	**ε [kJ/mol]**
**H (in C-O-H)** [a]	0.040001	0.19246
**O (in C-O-H)** [a]	0.302906	0.63639
**C (in C-O-H)** [b]	0.321400	0.23633
**C** [b]	0.321400	0.23633

[a] Adapted from Ref. [26]; [b] From Ref. [29].

Table S4. LJ Params for the diamond crystal terminated with H and OH groups.

**Atom type**	**σ [nm]**	**ε [kJ/mol]**
**H (in C-O-H)** [a]	0.040001	0.19246
**O (in C-O-H)** [a]	0.302906	0.63639
**C (in C-O-H)** [b]	0.321400	0.23633
**H (in C-H)** [a]	0.235200	0.09205
**C (in C-H)** [b]	0.321400	0.23633
**C** [b]	0.321400	0.23633

[a] Adapted from Ref. [26]; [b] From Ref. [29].

Boron nitride. The structure of hBN is hexagonal with the B and N atoms kept in fixed positions throughout the entire MD simulations. The experimental BN covalent bond distance is ~1.46 A. However, in order to make the system periodic along the *y*-and *x*-directions, we stretched slightly the hBN monolayer depending on the diamond surface considered (which is periodic along the *y*-and *x*-directions). Figure S9a shows the

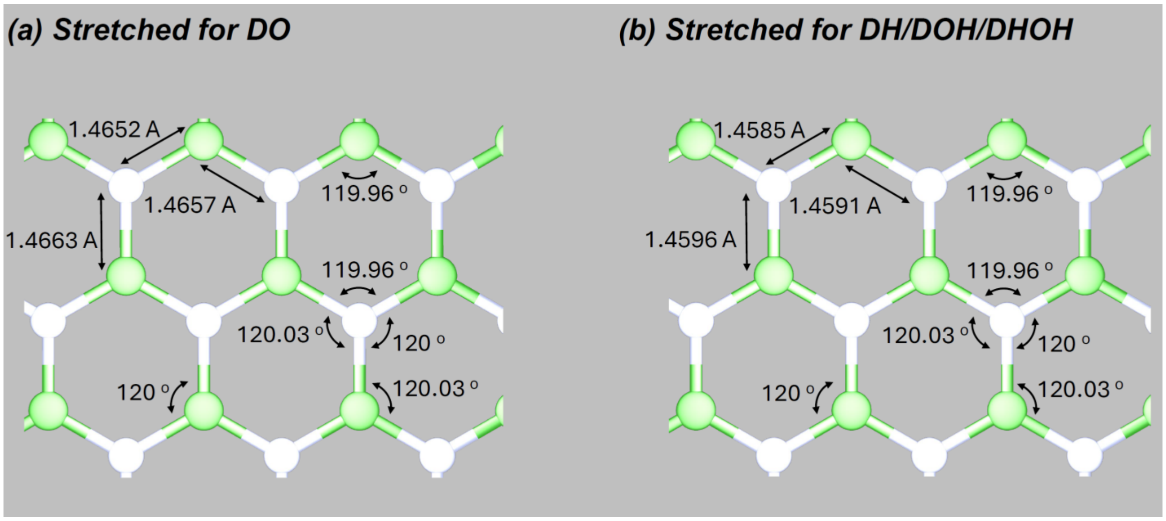

Fig. S9 | Structure of the hBN monolayer. The bond lengths and angles are indicated. Partial charges and LJ interaction parameters are given in Table S5.

geometry of the hBN monolayer in the presence of the diamond crystal terminated with O atoms. The geometry of hBN in the presence of the diamond crystal terminated with H, OH, and H+OH groups is shown in Fig. S9b. The N/B atoms interact with the water molecules and anions via electrostatic and LJ interactions; partial charges and LJ interaction parameters are given in Table S5. The Lorentz-Berthelot combination rules are used to generate the LJ parameters among the hBN atoms and the anions/water atoms.

Table S5. LJ parameters and partial charges for hBN.

Atom type	σ [nm]	ε [kJ/mol]	q [e]
**B** [a]	0.330870	0.28970	0.907
**N** [a]	0.321740	0.19790	-0.907

[a] From Ref. [30].

III.3 *Impact of the charge distribution on the diamond surface*

The results reported in the main text are based on a confined water system containing $N_{\mathrm{q}} = 2704$ anions (fluoride). As explained below, to compensate for the negative charges added to the confined water, we also *add* a positive charge to the C atoms located in the monolayer closest to the water volume. These C atoms form a covalent bond with the surface O atoms and are shown in blue in Fig. 4d of the main text; see also Fig. S10a below. In the case of the diamond surface terminated with OH groups, there are $N_{\mathrm{c}} = N_{\mathrm{q}}$ atoms in the C monolayer closest to water. Hence, each of these C atoms (shown in blue in Fig. S10a) are assigned an additional charge $q' = -q > 0$. Below, we study how the results from our MD simulations vary when the net positive charge in diamond is distributed uniformly over *all* the C atoms of the diamond crystal.

Figure S10a shows a snapshot of the same system studied in the main text (Fig. 4c-e; briefly, System 1) but with the compensating positive charge of diamond distributed over *all* C atoms of the diamond crystal, as explained above (briefly, System 2). Figure S10b shows the density profiles of the F^- ions and water taken from the main text (Fig. 4c; System 1) and the corresponding density profiles when the compensating positive charge is distributed over *all* C atoms in the diamond (System 2). The density profiles of Systems 1 and 2 practically overlap with one another. Figure S10c shows the fraction of water molecules in layers L1, L2, and L3 as a function of time. Again, these distributions are practically identical for Systems 1 and 2. Therefore, our results are insensitive to the specific distribution of the added positive charge over the diamond crystal.

The results in Figs. S10b and S10c can be understood by approximating each C monolayer within the diamond crystal with a homogeneously charged, infinite plane. Since an infinite charged plane generates a constant electric field (perpendicular to the plane), the same net electric field is generated if the compensating $N_{\mathrm{q}}|q|$ charge in diamond is placed in a single C monolayer or multiple C monolayers.

III.4 *Impact of diamond surface termination*

The results presented in Figs. 4c-e of the main text are based on a system comprising $N = 27738$ water molecules

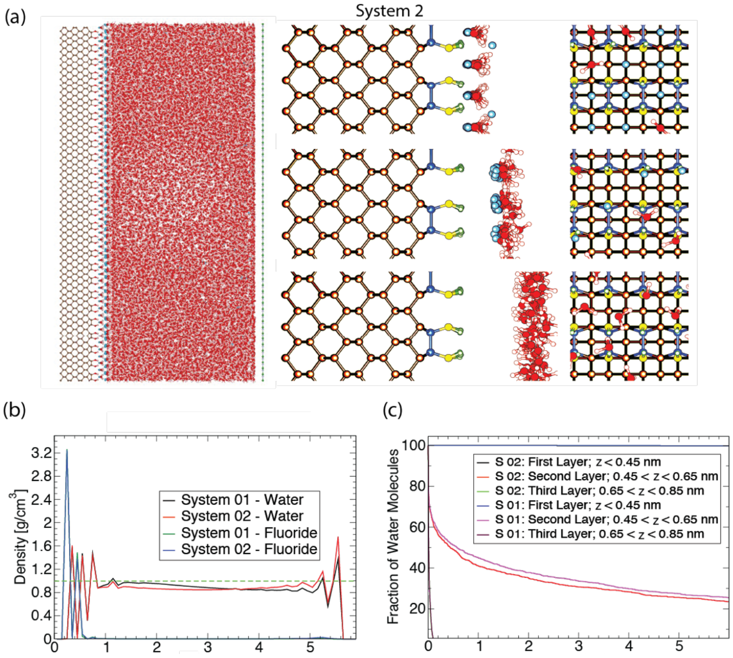

Fig. S10 | Impact of the positive charge distribution in the diamond. (a) Snapshot taken from our MD simulations of water ($N_w = 27738$) and fluoride anions ($N_q = 2704$) confined by hBN and diamond passivated with OH atoms. The hBN/diamond separation is $d \sim 5.7$ nm and $T = 300$ K (same as in Fig. 4a of the main text; briefly, System 1). To make the system charge-neutral, a net positive charge is distributed evenly over *all* C atoms of the diamond crystal (shown in blue and brown, denoted System 2). (b) Water and fluoride density profiles for Systems 1 and 2. (c) Fraction of water molecules that are initially in layers L1, L2, L3 and remain in the same layer at time t. The results in (b) and (c) are practically identical for Systems 1 and 2.

and $N_q = 2704$ fluoride atoms confined by hBN and hydroxylated diamond. Similar results hold when diamond is terminated with (i) O, (ii) H, and (iii) both H and OH groups. To show this, we include in Figs. S11, S12, and S13 the density profiles for cases (i), (ii), and (iii), respectively. Also included is the fraction of water molecules that are initially in the first, second, and third layers next to the diamond crystal that remain in the corresponding layer at time t (results for the hydroxylated diamond surface are in Fig. S10c). In all cases, the fluoride anions are located preferentially next to the diamond surface. The water molecules and fluoride ions next to the diamond surface are found at specific locations on the diamond structure, forming a square lattice. In this lattice, the fluoride ions and the first monolayer of water molecules are initially placed in a disordered manner, next to one another. Importantly, these water molecules and anions are unable to diffuse (cases (i) and (ii); see Figs. S11d and S12d) or have a very small diffusivity (case (iii); see Fig. S13d).

I.5 *Nanoconfined water (no anions added)*

As discussed in the main text (Figs. 4a-b), the layer of non-diffusive water molecules next to the diamond surface is no longer present when the fluoride anions are removed. Under these conditions, water remains in the liquid phase with no signs of vapor domains (see Fig. 4a of the main text, reproduced in Fig. S14a below). While water molecules still form layers next to the diamond surface and hBN (see Fig. 4b of the main text, reproduced in Fig. S14c below), these

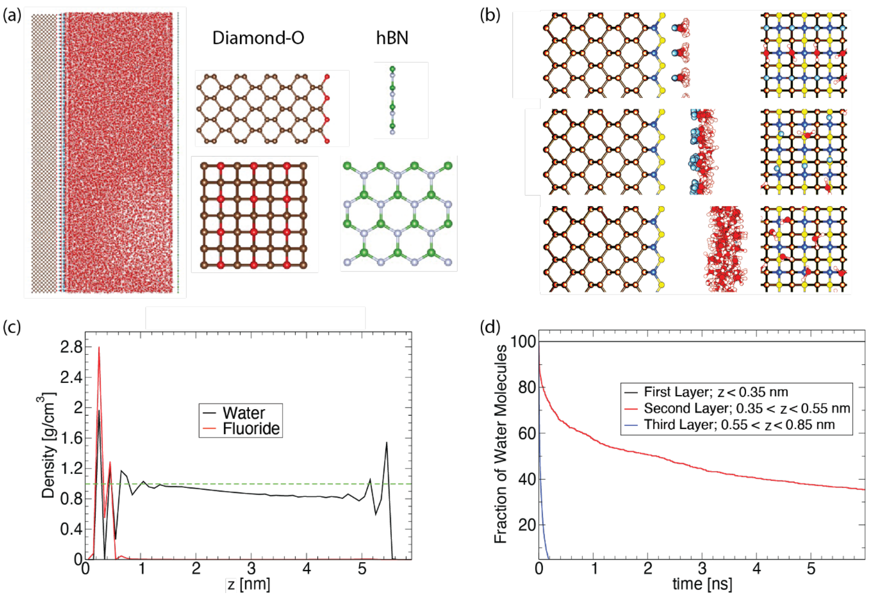

Fig. S11 | O termination. Snapshot from MD simulations of a system comprising $N_w = 27738$ water molecules and $N_q = 2704$ fluoride anions confined by an hBN monolayer and a diamond crystal passivated with O atoms. (b) Side and top view of the fluoride and water molecules located in the first (L1; top), second (L2, middle) and third (L3, bottom) water layers formed next to the diamond surface. (c) Water density profile as a function of the distance z from the diamond surface showing three water layers (L1, L2, L3) next to the diamond surface. (d) Fraction of water molecules that are initially in layers L1, L2, and L3 that remain in the corresponding layer at time t. Water layers located in layer L1 are unable to diffuse.

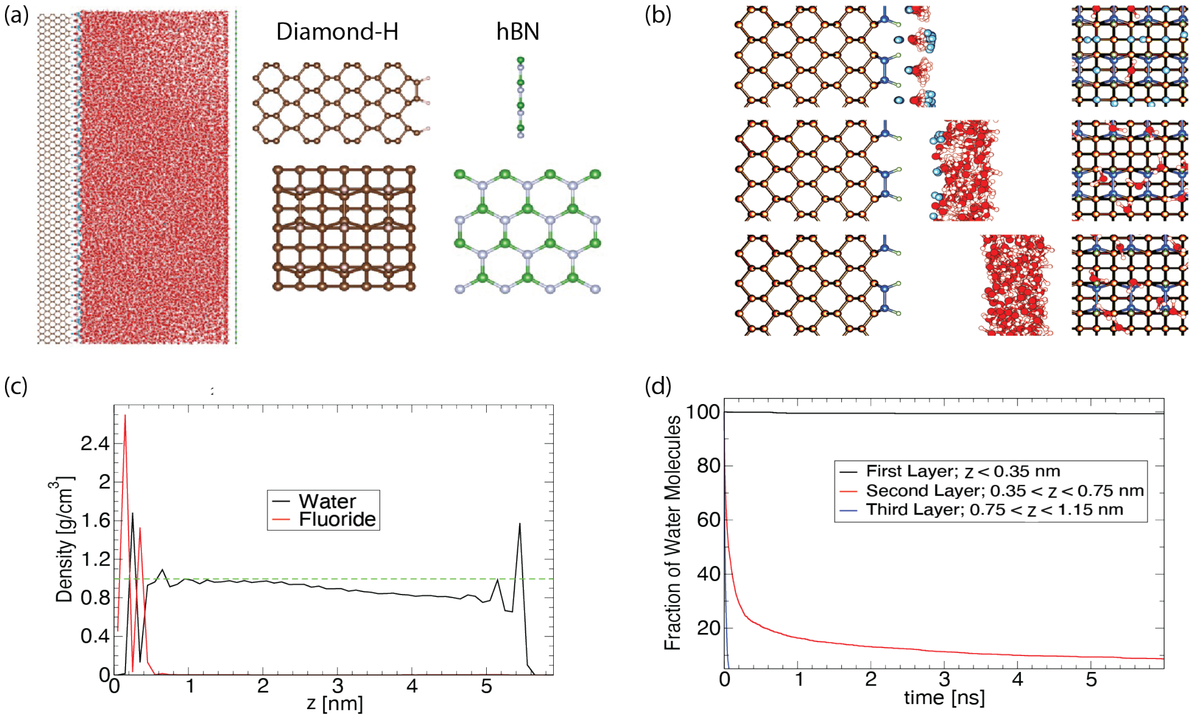

Figs. S12 | H termination. Same as in Fig. S11 for the case where the diamond surface is passivated with H atoms.

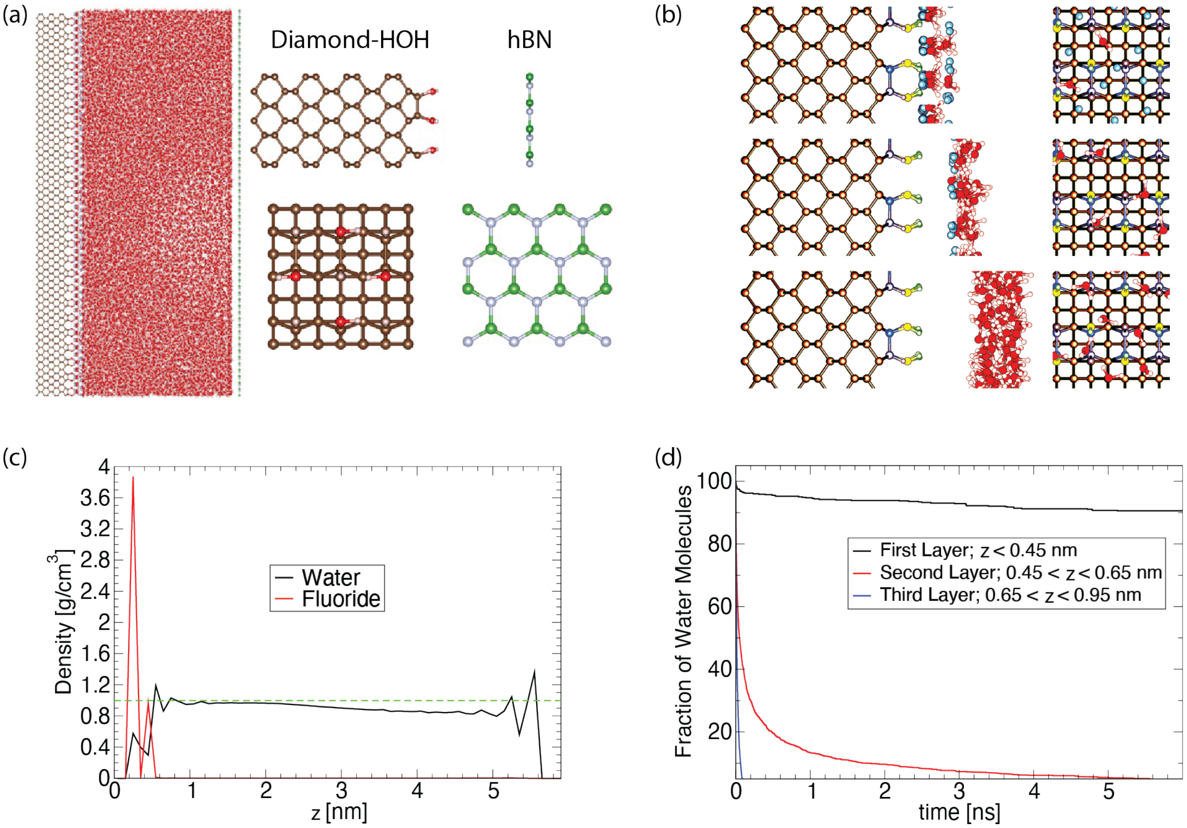

Figs. S13 | H and OH termination. Same as in Fig. S11 for the case where the diamond surface is passivated with H and OH groups.

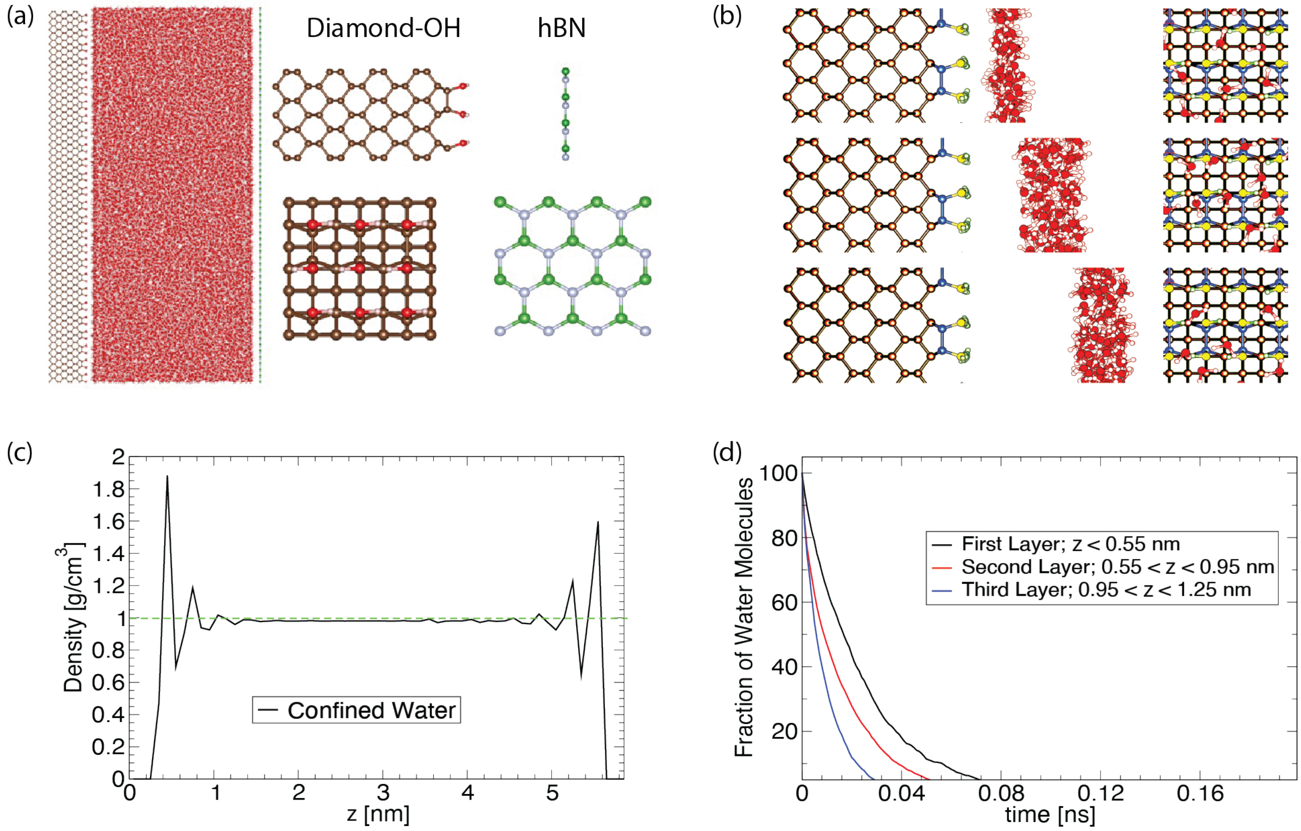

Fig. S14 | Dynamics of pure water. (a) Snapshot from MD simulations of water confined by an hBN monolayer, and diamond terminated with OH groups ($N = 30442$). The diamond crystal and hBN monolayer, as well as the diamond-hBN separation are identical to the system studied in Fig. 4 of the main text; $T = 300$ K. (b) Side and top view of the water molecules located in the first (L1; top), second (L2, middle) and third (L3, bottom) water layers formed next to the diamond surface. (c) Water density profile as a function of the distance z from the diamond surface showing the three water layers (L1, L2, L3) formed next to the diamond surface. At approximately >1 nm away from the diamond/hBN, water exhibits bulk-like properties; the density of water in this region is practically identical to the density of bulk (TIP4P/2005) water at $P = 0.1$ MP and $T = 300$ K (dashed green line). (d) Average fraction of water molecules in layers L1, L2, L3 at time $t = 0$ (next to the diamond surface) that remain in the same layer at time t. Layers L1, L2, L3 are defined from (c) and correspond to $z < 0.55$ nm, $0.55 < z < 0.95$ nm, and $0.95 < z < 1.25$ nm, respectively.

molecules can diffuse into the confined volume, and *vice versa*. This is indicated by (i) the mean-square displacement of the water molecules included in Fig. 4b of the main text, and (ii) by the fraction of water molecules in layers 1, 2, 3 at time $t = 0$ (next to the diamond surface) that remain in the same layer at time t (Fig. S14d).

III.6 *Impact of the number of anions in the confined water*

To explore the effect of increasing the number of anions in the confined water, we perform MD simulations for the same system studied in the main text but with twice the number of fluorides in the system ($N_q = 5408$, Fig. S15a). As explained in the main text, the additional negative charge is compensated by adding a positive charge to the C monolayer of diamond closest to the water volume (blue C atoms in Fig. S15b). As shown in Fig. S15b-c, the anions are located preferentially next to the diamond surface and are surrounded by water molecules. The water molecules and anions next to the surface are located on a square lattice, as in the original system (compare Fig. 4d of the main text and Fig. S15b below); these water molecules are not able to diffuse (Fig. S15d). In this regard, our results are insensitive to the number of fluoride anions in the system ($N_{F^-} = 2704 - 5408$). The main effect of adding anions seems to be to alter the layering of water molecules next to the diamond surface. Specifically, as shown in Figs. S15b and S15c below, adding anions leads to an accumulation of negative charges at $0.40 < z < 0.6$ nm. Water molecules at these distances are mostly excluded.

III.7 *Impact of anions size*

Our model system is based on classical MD simulations, and one may wonder if the results based on fluoride are sensitive to the anions size. From a classical perspective, an electron is represented by a point charge, much smaller and

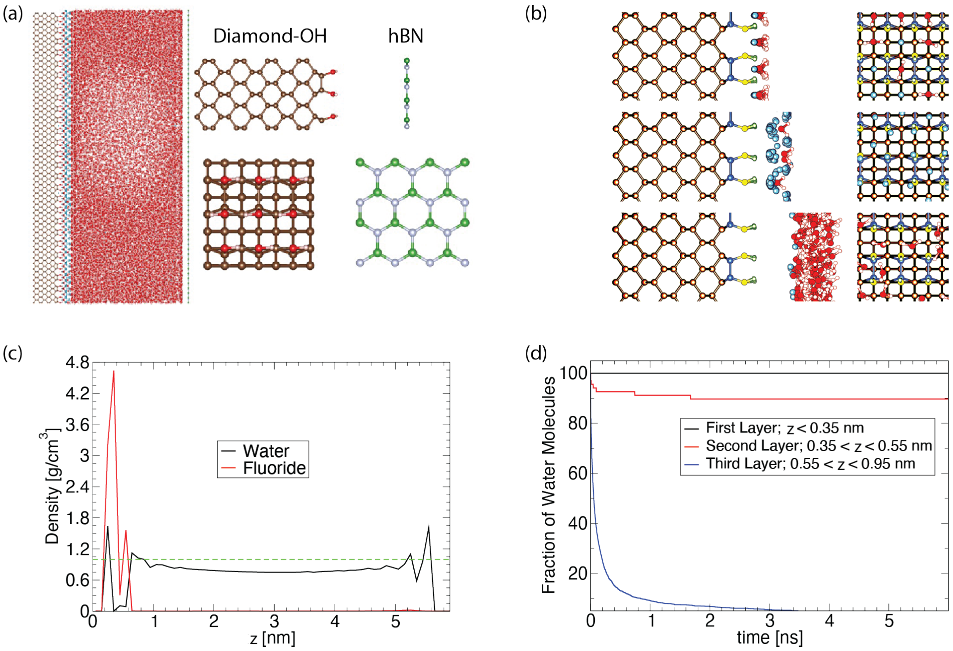

Fig. S15 | Impact of the number of anions. (a) Snapshot from MD simulations of a system comprising $N_w = 25034$ water molecules and $N_q = 5408$ fluoride anions confined by an hBN monolayer and a hydroxylated diamond crystal. (b) Side and top view of the fluoride and water molecules located in regions L1 (first water layer L1, $z < 0.35$ nm; top panel), L2 ($0.35 < z < 0.55$ nm; middle panel) and L3 (second water layer, $0.55 < z < 0.95$ nm; bottom panel). (c) Water density profile as a function of the distance z from the diamond surface showing the three regions L1, L2, and L3 close to the diamond surface. (d) Fraction of water molecules that are initially in regions L1, L2, and L3 that remain in the corresponding region at time t. Water layers located in layer L1 are unable to diffuse.

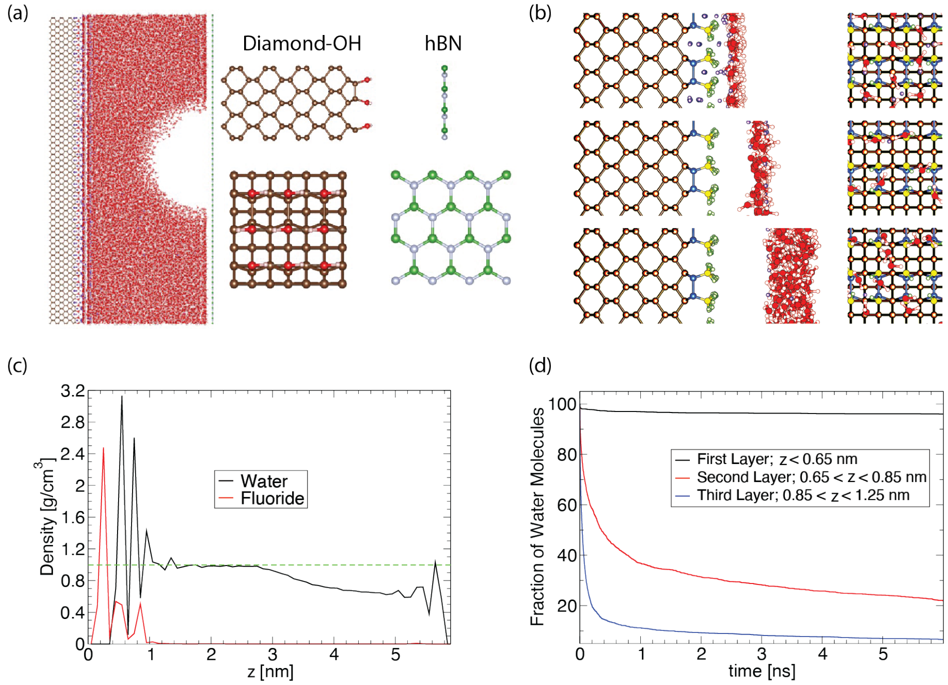

Fig. S16 | Impact of the anion size. Same system considered in Fig. 4c-e of the main text when the mass of the F- anions is reduced by 94.69% (from $m_F = 18.998$ amu $m' = 1.008$ amu) and the Lennard-Jones parameter σ is reduced by 30 % (from $\sigma_F = 0.379$ nm to $\sigma' = 0.265$ nm). (a) Snapshot of the system from the MD simulations. (b) Side and top view of the fluoride and water molecules located in the first ($z < 0.65$ nm, L1; top panel), second ($0.65 < z < 0.85$ nm, L2; middle panel), and third water layer ($0.85 < z < 1.25$ nm, L3; bottom panel). (c) Density profile as a function of the distance z from the diamond surface showing the three regions L1, L2, and L3 close to the diamond surface. (d) Fraction of water molecules that are initially in water layers L1, L2, and L3 that remain in the corresponding region at time t.

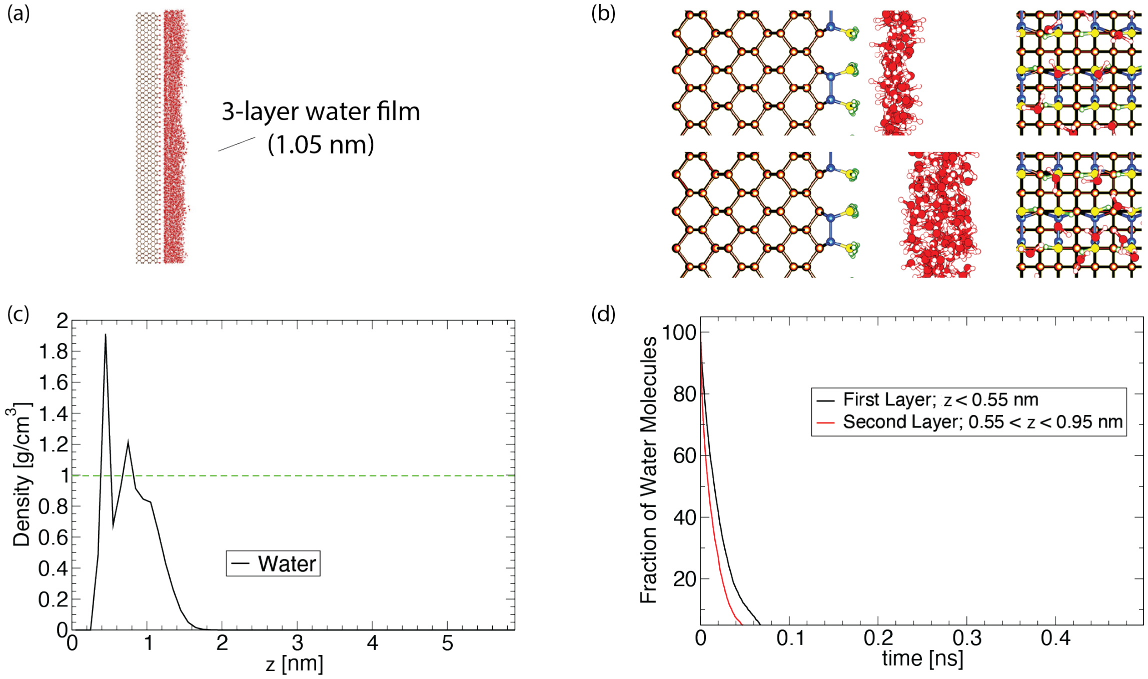

Fig. S17 | Dynamics of a pure water film. Snapshot from a MD simulation of a thin water film (approximately 1.0 nm-thick, $N = 4177$ water molecules) on the hydroxylated diamond surface; $T = 300$ K. (b) Density profile of water showing that water molecules arrange in two water layers. (c) Snapshots of the system showing the water molecules in the first ($z < 0.55$ nm; top panel) and second ($z > 0.55$ nm; bollom panel) layers. (d) Fraction of water molecules initially in layers 1 and 2 that remain in the corresponding layer at time t. This fraction decays to zero within < 100 ps indicating that the water molecules in both layers are able to diffuse over time.

lighter than fluoride (while the effects due to the electron delocalization should play an important role, such effects cannot be included in our classical model system). Here, we compare the results reported in the main text when the fluoride mass is reduced by 94.7% (from $m_F = 18.998$ amu $m' = 1.008$ amu) and the Lennard-Jones (LJ) parameter σ, which defines the anion size, is reduced by 30 % (from $\sigma_F = 0.379$ nm to $\sigma' = 0.265$ nm); the anion charge and LJ parameter ε are left unchanged. Our results based on the modified fluoride anions are shown in Fig. S16. At the diamond-hBN separation studied, and for the given number of water and anions, a pronounced cavitation develops within the system (Fig. S16a). This is because of two effects: (i) The smaller modified anions move deeper within the groves of the diamond surface which (ii) allows water molecules to locate closer to the diamond surface (Fig. S16b-c). Both (i) and (ii) create additional space for the liquid to fill, hence, forcing the liquid to cavitate. Interestingly, despite the cavitation (next to the hBN monolayer), the molecules in the first water monolayer seem to be unable to diffuse (Fig. S16d), as found in the main text for the case of water with fluoride anions.

III.8 *Thin water film*

Partial cavitation or incomplete filling of the nanopore could, in principle, also lead to the slowdown of water molecules. For example, cavitation could lead to the formation of thin films on the confining surfaces, and water molecules in such films could show low or negligible dynamics. To explore this possibility, we also perform MD simulations of a thin water film on the hydroxylated diamond surfaces (Fig. S17a). The water film is ~1 nm-thick and is composed of $N = 4177$ water molecules. After a few picoseconds, water molecules rearrange into two water layers (Fig. S17b); snapshots of water molecules in each layer are shown in Fig. S17c. We find that the water molecules in both layers are able to diffuse. To show this, we include in Fig. S17d the fraction of water molecules in layers $i = 1,2$ at a time $t = 0$ that remain in layer i as function of time. For both layers, we find that the fraction drops to nearly zero for $t < 100$ ps indicating that, indeed, water molecules diffuse over time.

References

[1] A. Bhardwaj, M.V.S. Martins, Y. You, R. Sajja, M. Rimmer, S. Goutham, R. Qi, S.A. Dar, B. Radha, A. Keerthi, "Fabrication of angstrom-scale two-dimensional channels for mass transport", *Nat. Prot.* **19**, 240 (2024).

[2] R. Frisenda, E. Navarro-Moratalla, P. Gant, D. Pérez De Lara, P. Jarillo-Herrero, R.V. Gorbachev, A. Castellanos-Gomez, "Recent progress in the assembly of nanodevices and van der Waals heterostructures by deterministic placement of 2D materials", *Chem. Soc. Rev.* **47**, 53 (2018).

[3] J. Henshaw, P. Kehayias, M. Saleh Ziabari, M. Titze, E. Morissette, K. Watanabe, T. Taniguchi, J.I.A. Li, V.M. Acosta, E.S. Bielejec, M.P. Lilly, A.M. Mounce, "Nanoscale solid-state nuclear quadrupole resonance spectroscopy using depth-optimized nitrogen-vacancy ensembles in diamond", *Appl. Phys. Lett.* **120**, 174002 (2022).

[4] T. Staudacher, F. Shi, S. Pezzagna, J. Meijer, J. Du, C.A. Meriles, F. Reinhard, J. Wrachtrup, "Nuclear magnetic resonance spectroscopy on a (5nm)^3 volume of liquid and solid samples", *Science* **339**, 561 (2013).

[5] A. Laraoui, F. Dolde, C. Burk, F. Reinhard, J. Wrachtrup, C.A. Meriles, "High-resolution correlation spectroscopy of ^{13}C spins near a nitrogen-vacancy center in diamond", *Nat. Commun.* **4**, 1651 (2013).

[6] T.M. Staudacher, N. Raatz, S. Pezzagna, J. Meijer, F. Reinhard, C.A. Meriles, J. Wrachtrup, "Probing molecular dynamics at the nanoscale via an individual paramagnetic center", *Nat. Commun.* **6**, 8527 (2015).

[7] C.A. Meriles, J. Liang, G. Goldstein, P. Cappellaro, J. Hodges, J. Maze, M.D. Lukin, "Imaging mesoscopic nuclear spin noise with a diamond magnetometer", *J. Chem. Phys.* **133**, 124105 (2010).

[8] N. Staudenmaier, A. Vijayakumar-Sreeja, S. Oviedo-Casado, G. Genov, D. Cohen, D. Dulog, T. Unden, N. Striegler, A. Marshall, J. Scheuer, C. Findler, J. Lang, I. Schwartz, P. Neumann, A. Retzker, F. Jelezko, "Power-law scaling of correlations in statistically polarised nano-NMR", *npj Quant. Inf.* **8**, 120 (2022).

[9] K. Xu, D. Pagliero, G.I. Lopez Morales, J. Flick, A. Wolcott, and Carlos A. Meriles, "Photoinduced charge injection from shallow point defects in diamond into water", *ACS Appl. Mater. Interf.* **16**, 37226 (2024).

[10] A. Chemin, I. Levine, M. Rusu, R. Vaujour, P. Knittel, P. Reinke, K. Hinrichs, T. Unold, T. Dittrich, T. Petit, "Surface-mediated charge transfer of photogenerated carriers in diamond", *Small Meth.* 2300423 (2023).

[11] X. Gao, L. Liu, D. Qi, S. Chen, A.T.S. Wee, T. Ouyang, K.P. Loh, X. Yu, H.O. Moser, "Water-induced negative electron affinity on diamond (100)", *J. Phys. Chem. C* **112**, 2487 (2008).

[12] A. Stacey, N. Dontschuk, J-P. Chou, DA. Broadway, A.K. Schenk, M.J. Sear, J-P. Tetienne, A. Hoffman, S. Prawer, C.I. Pakes, A. Tadich, N.P. de Leon, A. Gali, L.C. L. Hollenberg, "Evidence for primal sp2 defects at the diamond surface: candidates for electron trapping and noise sources", *Adv. Mater. Interfaces* **6**, 1801449 (2019)

[13] M.V. Hauf, B. Grotz, B. Naydenov, M. Danker, S. Pezzagna, J. Meijer, F. Jelezko, J. Wrachtrup, M. Stutzmann, F. Reinhard, J.A. Garrido, "Chemical control of the charge state of nitrogen-vacancy centers in diamond", *Phys. Rev. B* **83**, 081304(R) (2011)

[14] D. Zhu, L.H. Zhang, R.E. Ruther, R.J. Hamers, "Photo-illuminated diamond as a solid-state source of solvated electrons in water for nitrogen reduction", *Nat. Mater.* **12**, 836 (2013).

[15] F. Buchner, T. Kirschbaum, A. Venerosy, H. Girard, J-C. Arnault, B. Kiendl, A. Krueger, A. Bande, T. Petit C. Merschjann, K. Larsson, "Early dynamics of the emission of solvated electrons from nanodiamonds in water", *Nanoscale* **14**, 17188 (2022).

[16] Z. Luz, R.G. Shulman, "Proton magnetic resonance shifts in aqueous solutions of paramagnetic metal ions", *J. Chem. Phys.* **43**, 3750 (1965).

[17] "NV$^-$ with nitrogen", N.B. Manson, M.S.J. Barson, M. Hedges, P. Singh, S. Stearn, Y-H. Chen, L. Oberg, C.A. Meriles, M.W. Doherty, arXiv:2311.09570v1 [cond-mat.mtrl-sci], 16 Nov 2023, https://doi.org/10.48550/arXiv.2311.09570 (accessed 2023-11-16).

[18] M.E. Tuckerman, D. Marx, M. Parrinello, "The nature and transport mechanism of hydrated hydroxide ions in aqueous solution", *Nature* **417**, 925 (2002).

[19] J.L.F. Abascal, C. Vega, "A general purpose model for the condensed phases of water: TIP4P/2005", *J. Chem. Phys.* **123**, 234505 (2005).

[20] https://doi.org/10.5281/zenodo.8134358.

[21] S. Nosé, "A molecular dynamics method for simulations in the canonical ensemble", *Mol. Phys.* **52**, 255 (1984).

[22] W. G. Hoover, "Canonical dynamics: Equilibrium phase-space distributions", *Phys. Rev. A* **31**, 1695 (1985).

[23] U. Essmann, L. Perera, M.L. Berkowitz, T. Darden, H. Lee, L.G. Pedersen, "A smooth particle mesh Ewald method", *J. Chem. Phys.* **103**, 8577 (1995).

[24] J. Wong, D.A. Jahn, N. Giovambattista, "Pressure-induced transformations in glassy water: A computer simulation study using the TIP4P/2005 model", *J. Chem. Phys* **143**, 074501 (2015).

[25] S. Blazquez, M.M. Conde, J.L.F. Abascal, C. Vega, "The Madrid-2019 force field for electrolytes in water using TIP4P/2005 and scaled charges: Extension to the ions F^-, Br^-, I^-, Rb^+, and Cs^+", *J. Chem. Phys.*156, 044505 (2022).

[26] S. Reiling, M. Schlenkrich, J. Brickmann, "Force field parameters for carbohydrates", *J. Comput. Chem.* **17**, 450 (1996).

[27] M. Kaviani, P. Deák, B. Aradi, T. Frauenheim, J.-P. Chou, A. Gali, "Proper surface termination for luminescent near-surface nv centers in diamond", *Nano Lett.* **14**, 4772 (2014).

[28] S.J. Sque, R. Jones, P.R. Briddon, "Structure, electronics, and interaction of hydrogen and oxygen on diamond surfaces", *Phys. Rev. B* **73**, 085313 (2006).

[29] T. Werder, J.H. Walther, R.L. Jaffe, T. Halicioglu, P. Koumoutsakos, "On the water−carbon interaction for use in molecular dynamics simulations of graphite and carbon nanotubes", *J. Phys. Chem. B* **107**, 1345 (2003).

[30] A.G. Rajan, M.S. Strano, D. Blankschtein, "Ab initio molecular dynamics and lattice dynamics-based force field for modeling hexagonal boron nitride in mechanical and interfacial applications", *J. Chem. Phys. Lett.* **9**, 1584 (2018).
